# Gut-specific histamine 3 receptor signaling orchestrates microglia-dependent resolution of peripheral inflammation

**DOI:** 10.1172/JCI184697

**Published:** 2025-07-10

**Authors:** Kerstin Dürholz, Leona Ehnes, Mathias Linnerbauer, Eva Schmid, Heike Danzer, Michael Hinzpeter-Schmidt, Lena Lößlein, Lena Amend, Michael Frech, Vugar Azizov, Fabian Schälter, Arne Gessner, Sébastien Lucas, Till-Robin Lesker, R. Verena Taudte, Jörg Hofmann, Felix Beyer, Hadar Bootz-Maoz, Yasmin Reich, Hadar Romano, Daniele Mauro, Ruth Beckervordersandforth, Maja Skov Kragsnaes, Torkell Ellingsen, Wei Xiang, Aiden Haghikia, Cezmi A. Akdis, Francesco Ciccia, Tobias Bäuerle, Kerstin Sarter, Till Strowig, Nissan Yissachar, Georg Schett, Veit Rothhammer, Mario M. Zaiss

**Affiliations:** 1 Department of Internal Medicine 3, Rheumatology and Immunology,; 2 Deutsches Zentrum Immuntherapie (DZI), and; 3 Department of Neurology, Universitätsklinikum Erlangen, Friedrich-Alexander-Universität Erlangen-Nürnberg, Erlangen, Germany.; 4 Department of Microbial Immune Regulation, Helmholtz Centre for Infection Research, Braunschweig, Germany; Hannover Medical School, Hannover, Germany.; 5 Institute of Experimental and Clinical Pharmacology and Toxicology, Friedrich-Alexander University Erlangen-Nürnberg, Erlangen, Germany.; 6 Core Facility for Metabolomics, Philipp University Marburg, Marburg, Germany.; 7 Department of Biology, Division of Biochemistry, Friedrich-Alexander University, Erlangen, Germany.; 8 Institute of Biochemistry, Friedrich-Alexander University of Erlangen-Nürnberg, Erlangen, Germany.; 9 The Goodman Faculty of Life Sciences, and Bar-Ilan Institute of Nanotechnology and Advanced Materials, Bar-Ilan University, Ramat-Gan, Israel.; 10 Department of Precision Medicine, University of Campania “Luigi Vanvitelli,” Naples, Italy.; 11 Department of Rheumatology, Odense University Hospital, Odense, Denmark.; 12 Department of Clinical Research, University of Southern Denmark, Odense, Denmark.; 13 Department of Molecular Neurology, University Hospital Erlangen, Friedrich-Alexander-Universität Erlangen-Nürnberg, Germany.; 14 Department of Molecular Cell Biology, Institute of Biochemistry and Pathobiochemistry, Ruhr University Bochum, Bochum, Germany.; 15 Swiss Institute of Allergy and Asthma Research, University of Zurich, Davos, Switzerland.; 16 Radiologisches Institut, Universitätsklinikum Erlangen, Friedrich-Alexander-Universität Erlangen-Nürnberg, Erlangen, Germany.

**Keywords:** Autoimmunity, Immunology, Autoimmune diseases, Rheumatology

## Abstract

Chronic inflammatory diseases like rheumatoid arthritis (RA) have been described to cause CNS activation. Less is known about environmental factors that enable the CNS to suppress peripheral inflammation in RA. Here, we identified gut microbiota–derived histamine as such a factor. We showed that low levels of histamine activate the enteric nervous system, increase inhibitory neurotransmitter concentrations in the spinal cord, and restore homeostatic microglia, thereby reducing inflammation in the joints. We found that elective histamine 3 receptor (H3R) signaling in the intestine was critical for this effect, as systemic and intrathecal application did not show effects. Microglia depletion or pharmacological silencing of local nerve fibers impaired oral H3R agonist–induced pro-resolving effects on arthritis. Moreover, therapeutic supplementation of the short-chain fatty acid propionate revealed one way to expand local intestinal histamine concentrations in mice and humans. Thus, we define a gut/CNS/joint axis pathway where microbiota-derived histamine initiates the resolution of arthritis via the CNS.

## Abstract 2

Gut microbiota-derived histamine activates the enteric nervous system, enhances spinal inhibitory neurotransmitters, and restores microglia, reducing arthritis inflammation via the gut-CNS-joint axis pathway.

## Graphical abstract

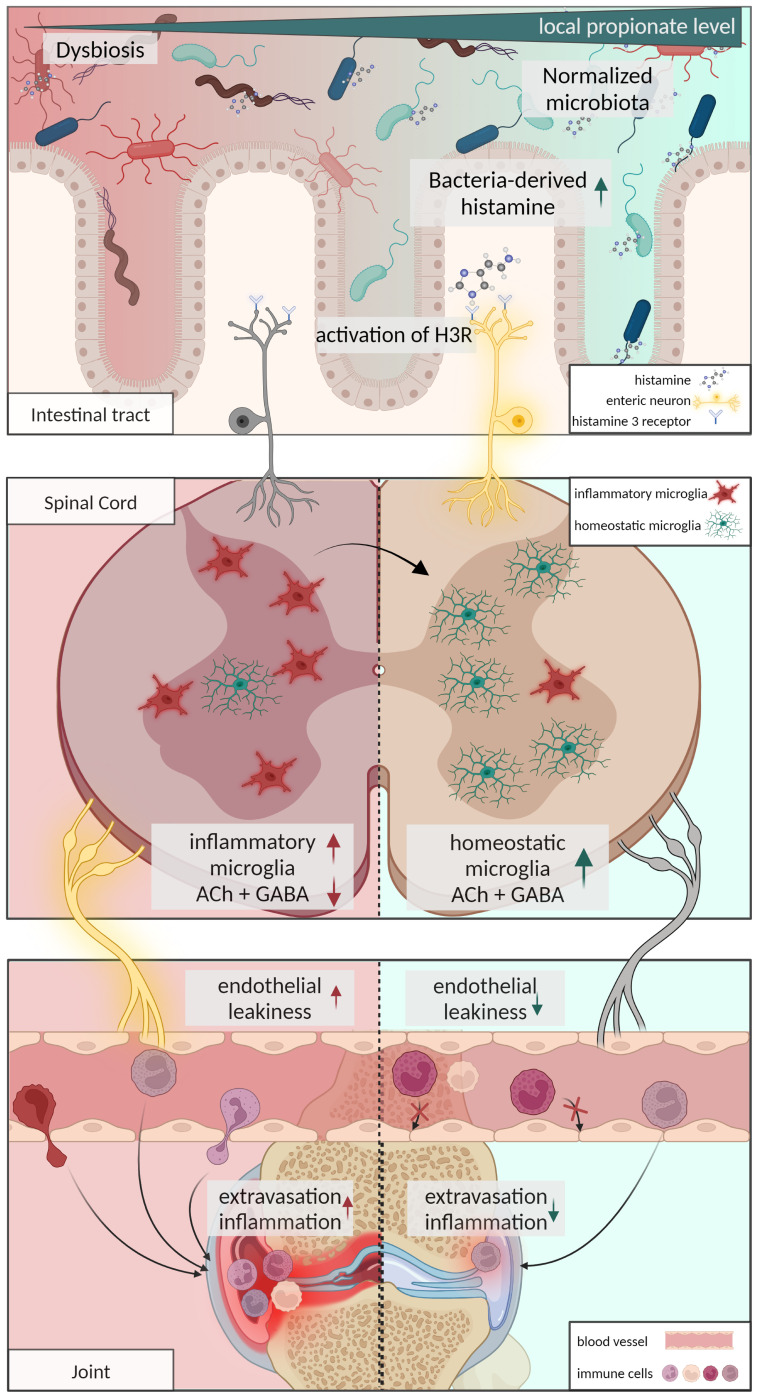

## Introduction

Rheumatoid arthritis (RA) is one of the most common and severe chronic inflammatory diseases with a lack of spontaneous resolution, which generally requires lifelong treatment with antirheumatic drugs. The disease is characterized by chronic synovial inflammation that leads to the destruction of the affected joints and increases disability (1). One essential step to initiate the resolution of arthritis is to prevent the influx of new cells via transendothelial migration from blood vessels into the inflamed synovium, while at the same time draining proinflammatory cells leave the affected joints. Earlier studies showed the potential impact of the CNS and sympathetic nerve fibers in control of blood flow and vascular permeability in the arthritic joints (2, 3) and its direct relevance for resolution (4, 5). However, little effort has been made to unravel how these processes are controlled and whether and how environmental factors, such as the microbiota, can initiate resolution of arthritis via the CNS; this is surprising given the vast literature on microbial dysbiosis (6, 7), the mucosal origin hypothesis (8), and the gut/joint axis (9) and its substantial role in RA.

Beyond joint pathology, RA is associated with neuropsychiatric comorbidities, including depression (10), anxiety (11), and an increased risk of developing neurodegenerative diseases in later life (12). However, the potential contribution of chronic pain to the development of depression and anxiety should not be overlooked. Preclinical studies from the early 1990s (13, 14) and 2000s (15–17) highlighted the potential for neuroimmune crosstalk in animal models for RA by showing that peripheral synovial inflammation is closely linked to the CNS.

In the CNS, the biogenic vasoactive amine histamine, which is known for its acute immediate hypersensitivity responses, also acts as a neurotransmitter in the histaminergic system. Histamine was shown to have pleiotropic effects on immune cells that are dependent on signaling via one of its 4 receptors (H1R–H4R). In preclinical models of arthritis, cellular-derived histamine was described as exerting proarthritic effects (18), mainly via H4R expression on hematopoietic cells (19, 20) and stimulation of osteoclastogenesis (21). In contrast, H3R is expressed by cells of the CNS and histaminergic neurons but also on intestinal endocrine cells (22), such as enterochromaffin cells (23) linking the gut to the CNS (24). Recent studies using human H3R ligands revealed antiinflammatory effects of H3R activation on neuroinflammation (25), matching reports of exacerbated neuroinflammatory disease in H3R knockout mice (26).

In addition to mammalian cells, bacteria can also secrete histamine following decarboxylation of histidine via the enzyme histidine decarboxylase (HDC) (27). However, the influence of microbiota-derived histamine on host immunological processes (i.e., the resolution of inflammation) is still poorly understood (28).

Here, we exploited untargeted metabolomics and mRNA-Seq of microglia from spinal cord tissues after oral histamine treatment in collagen-induced arthritis (CIA) or experimental autoimmune encephalomyelitis (EAE) mice to identify potential pro-resolving mediators of synovial and central inflammation; assessed their function in vivo using PLX5622-driven microglia or QX-314 plus bupivacaine-induced nerve fiber blockage; and extended these findings to functional magnetic resonance tomography analysis for vascular permeability in synovial tissues. The resulting data emphasize the importance of an acutely induced inflammatory microglial phenotype for arthritis persistence, which can be restored to a homeostatic microglial gene expression profile by microbiota-derived histamine, resulting in rapid resolution of synovial inflammation.

Taken together, these data highly contribute to our understanding of the gut/joint axis by implicating CNS microglial cells as a direct mediator of interorgan signaling, thereby providing alternative therapeutic options to resolve arthritis. Further, fiber-rich or short-chain fatty acid (SCFA) propionate treatments naturally increased microbiota-derived histamine levels in mice as well as patients with RA or multiple sclerosis (MS). These are important findings as they imply the value of minimally invasive treatments, such as diet and supplementation in resolution of inflammation.

## Results

### Propionate-induced microbial metabolites transfer pro-resolving effects.

**Figure 1:**
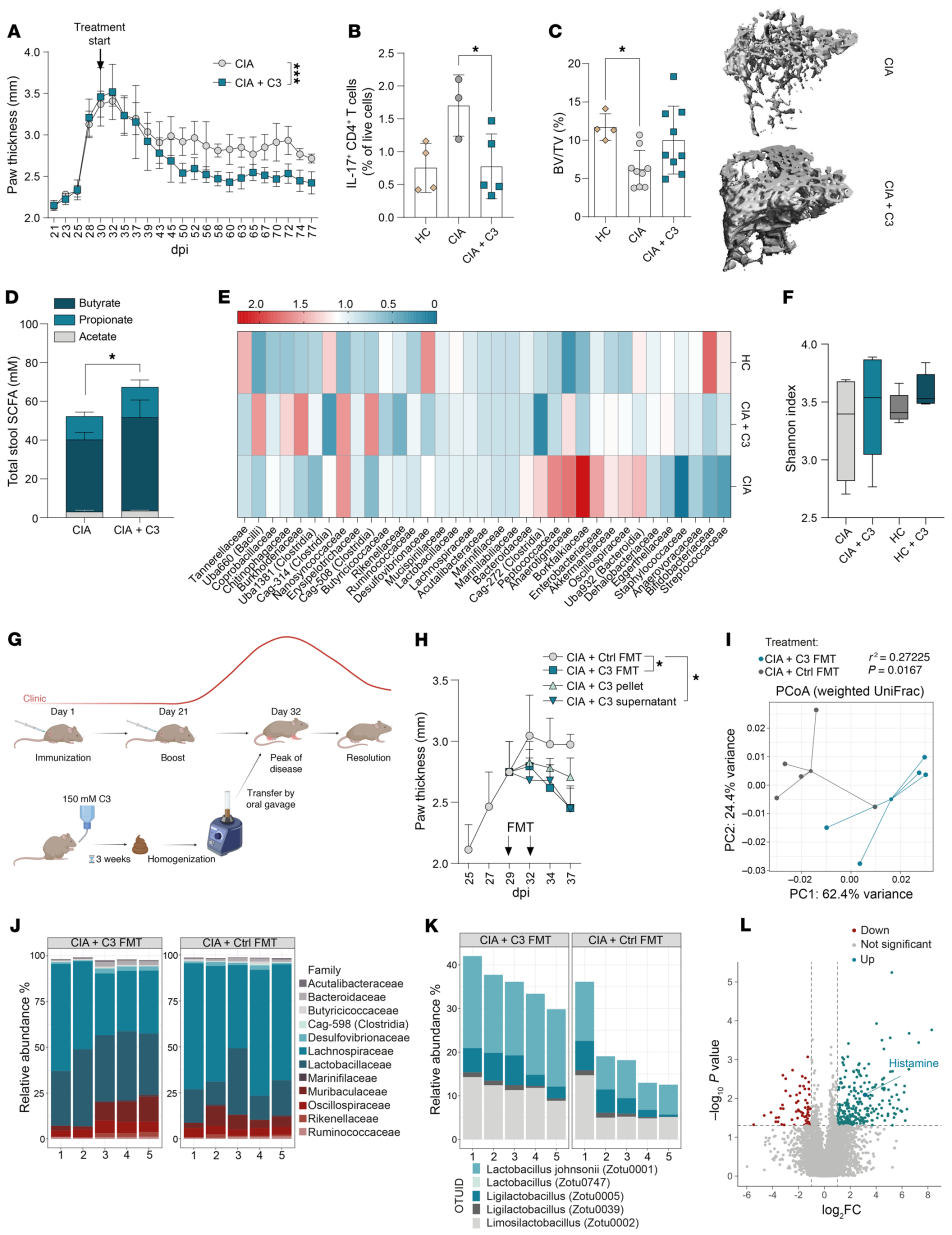
Propionate-induced microbial metabolites transfer pro-resolving effects. (**A**) Clinical arthritis score shown as paw thickness (mm) of CIA mice ± 150 mM C3 (*n* = 4–5) in drinking water starting at 30 dpi. (**B**) Flow cytometric analysis of IL-17^+^ CD4^+^ T cells in the spleen of healthy mice and CIA mice ± C3. (**C**) quantification of bone mass (BV/TV) and representative μCT images of the trabecular part of tibial bone of healthy mice and CIA mice ± C3, analyzed at 77 dpi. (**D**) SCFA levels in the cecum content of CIA mice ± C3. (**E**) 16s rRNA-Seq of the cecum content of healthy mice, CIA mice, and CIA mice ± C3. (**F**) Alpha-diversity measure of 16s rRNA-Seq data. (**G**) Experimental layout of FMT experiment. This overview was generated with BioRender. (**H**) Clinical arthritis score shown as paw thickness (mm) of CIA mice treated with FMT of naive donors, C3-treated donors, pellet of C3-treated donors, or supernatant of C3-treated donors. (**I**) PCoA plot of 16s rRNA-Seq of mice after control or C3 FMT. (**J**) Relative abundance of the bacterial families identified by 16s rRNA-Seq. Permutational multivariate analysis of variance (ADONIS) was significant (R^2^ = 0.27225, *P* = 0.0167). (**K**) Relative abundance of most strongly changed *Lactobacillaceae* strains. (**L**) Volcano plot of untargeted metabolomics analysis of stool supernatant fraction obtained from control versus C3-treated FMT donors (cutoffs: *P* < 0.05 and –1 < log_2_FC < 1). Data are expressed as mean ± SD. Statistical difference was determined by ADONIS (**L**), 1-way ANOVA (**B**, **C**, **F**), Student’s *t* test (**D**), and AUC (**A** and **H**). *P < 0.05, **P < 0.01, ***P < 0.001, ****P < 0.0001. CIA, collagen-induced arthritis; C3, propionate; BV, bone volume; TV, tissue volume; FMT, fecal microbiota transfer; PCoA, principal coordinates analysis.

Prophylactic nutritional fiber or propionate supplementation was shown to be effective in RA and MS animal models (29, 30). To study potential therapeutic effects, we supplemented the drinking water of CIA mice from the peak of disease at 30 days after immunization (dpi) with 150 mM propionate (C3) or high-fiber supplementation. Twenty days after C3 or high-fiber supplementation, CIA mice showed significantly improved clinical signs of arthritis (Figure 1A and Supplemental Figure 1, A–C; supplemental material available online with this article; https://doi.org/10.1172/JCI184697DS1), reduced splenic Th17 cells (Figure 1B and Supplemental Figure 1D), and restored systemic bone density compared with respective nontreated controls (Figure 1C). C3 treatment increased total SCFA concentrations in the intestine (Figure 1D) and reestablished a gut microbial composition similar to the preclinical phase in healthy controls by reducing previously identified arthritis-related species such as from the *Akkermansiaceae* (31) and *Enterobacteriaceae* (32) family while increasing the Shannon index over non-C3-treated CIA control mice (Figure 1, E and F). To address whether C3-modulated microbiota contributes to this finding, we established a fecal microbiota transfer (FMT) model in CIA mice (Figure 1G). To that end, FM donor DBA/1 mice were supplemented with C3 in their drinking water for 3 weeks. FM (C3 FM) was harvested and further divided into FM pellet (C3 pellet) and FM supernatant (C3 supernatant) after centrifugation. Similar to therapeutic C3 supplementation in drinking water, C3 FMT, when done at the peak of arthritis, promoted fast resolution compared with transfer of control FM (Figure 1H). When comparing the efficacy of the two treatments from the peak of the disease, the administration of C3 FM resulted in a significant improvement in arthritis scores after only 5 days, whereas the supplementation of C3 in drinking water only led to a significant improvement after 20 days. Within the 3 FMT groups, only C3 supernatant was as effective as complete C3 FM, whereas the C3 pellet fraction showed no statistical clinical improvement (Figure 1H). This finding was reflected in the flow cytometry analyses of spleen cells, which showed that splenic Th17 cells decreased fast after C3 FM treatment as well as after supplementation of C3 in the drinking water (Supplemental Figure 1E). Comparison of 16s rRNA bacterial community profiles after C3 FMT revealed clear differences in the β-diversity 5 days after transfer over FM controls (Figure 1I) and an enrichment in *Lactobacillaceae* accompanied by lower levels of *Lachnospiraceae* (Figure 1, J and K). 16s rRNA analysis revealed further enriched *Lactobacillaceae* in C3 supernatant over the C3 pellet group (Supplemental Figure 1F). The effectiveness of *Lactobacillaceae* as probiotics, especially for *Lactobacillus johnsonii*, which was increased in our experiments, has been shown in a variety of inflammatory diseases (33–35).

Because C3 supernatant FMT was equally effective as complete C3 FMT, we next performed untargeted metabolic analyses to identify possible effector molecules induced by C3 nutritional supplementation. Volcano plots of C3 supernatant FM identified significant upregulated and downregulated metabolites over FM control supernatant (Figure 1L). Next, to identify responsible effector molecules in C3 supernatant FM, size exclusion chromatography (SEC) was used to separate the supernatant into 5 subfractions with different ranges of molecular size. Strikingly, only C3 supernatant FM fraction number 1, containing only the smallest molecules, showed similar pro-resolving effects as unfractionated C3 supernatant FM (Supplemental Figure 1, G and H). Untargeted metabolomics analysis of the nonfractioned C3 supernatant identified histamine among the most upregulated metabolites, which is also part of the pro-resolving fraction 1, due to its small molecular weight of 111.15 Da (Figure 1K). Another study also showed a tendency for histamine levels to increase in the colon after C3 treatment (36). Taken together, all these observations suggest potent peripheral pro-resolving effector functions of intestinal histamine that is locally increased in the intestine after high-fiber or C3 dietary supplementation in mice.

### Resolution of arthritis depends on intestinal histamine and H3R signaling.

**Figure 2:**
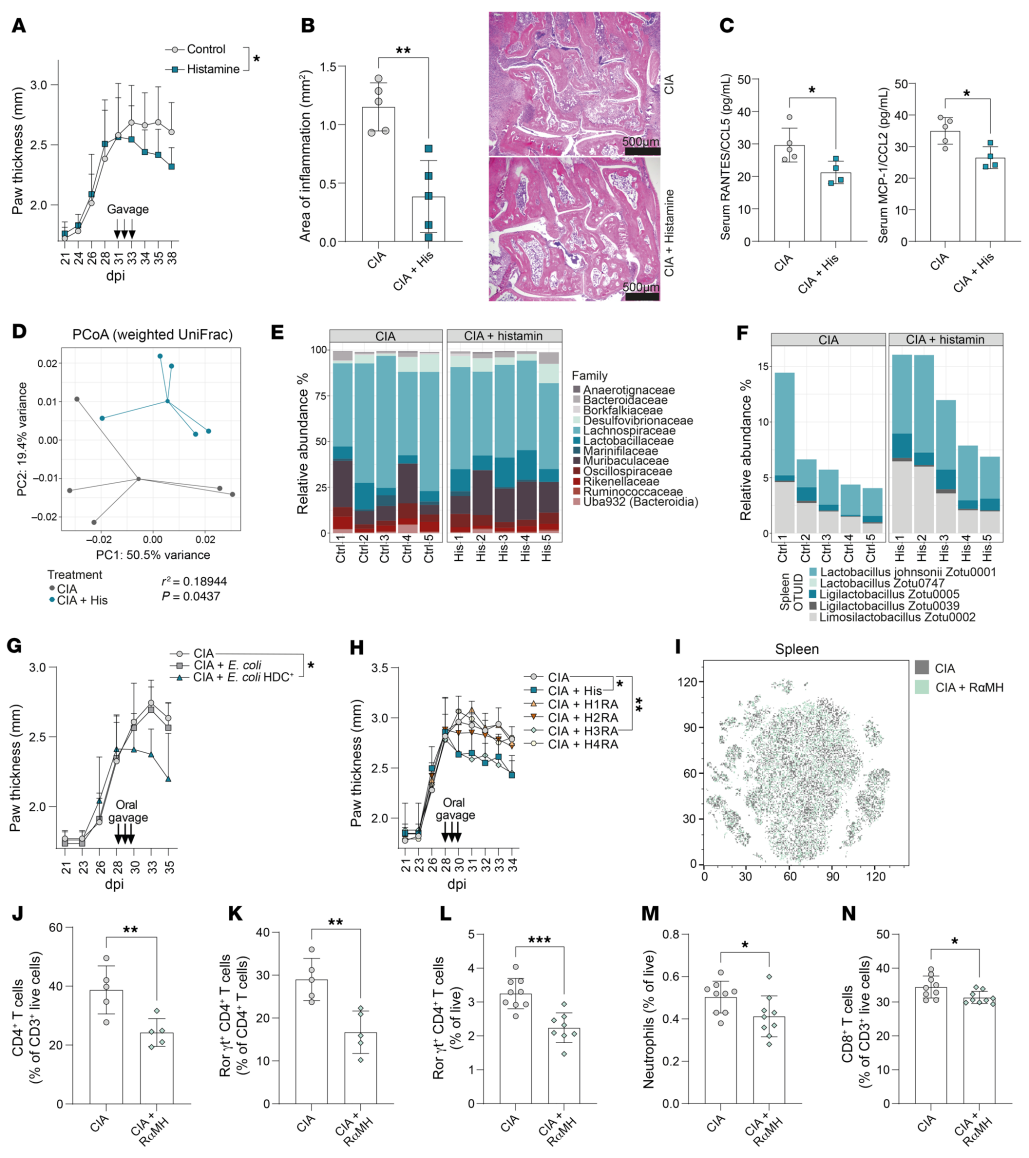
Resolution depends on intestinal histamine and H3R signaling. (**A**) Clinical arthritis score shown as paw thickness (mm) of CIA mice ± histamine (*n* = 12–14) at the peak of disease. (**B**) Area of inflammation expressed as absolute mm^2^ per analyzed H&E-stained paw sections of CIA mice ± histamine (*n* = 5) and example histology images. Scale bars: 500 μm. (**C**) Serum levels of RANTES/CCL5 and MCP-1/CCL2. (**D**) PCoA plot of 16s rRNA-Seq of CIA mice ± histamine. Permutational multivariate analysis of variance (ADONIS) was significant (R^2^ = 0.18944, *P* = 0.0437). (**E**) Relative abundance of the bacterial families identified by 16s rRNA-Seq. (**F**) Relative abundance of most changed *Lactobacillaceae* strains after histamine treatment. (**G**) Clinical arthritis score shown as paw thickness (mm) of CIA mice after transfer of PBS (control), *E*. *coli*, or HDC positive *E*. *coli* at the peak of disease (*n* = 4–5). (**H**) Clinical arthritis score shown as paw thickness (mm) of CIA mice after oral transfer of histamine or specific agonists for H1R–H4R. (*n* = 4–5). (**I**) t-SNE plot of spectral flow cytometric analysis of the spleen from CIA mice plus H3R agonist RαMH. (**J**) CD4^+^ T cells in the synovium. (**K**) RORγt^+^ CD4^+^ T cells in the synovium. (**L**) RORγt^+^ CD4^+^ T cells in the pLN. (**M**) Neutrophils in the pLN. (**N**) CD8^+^ T cells in the pLN. Data are expressed as mean ± SD. Statistical difference was determined by Student’s *t* test (**B**, **C**, and **J**–**N**), *t* test or 1-way ANOVA of AUC (**A**, **G**, and **H**), and ADONIS (**D**). *P < 0.05, **P < 0.01, ***P < 0.001, ****P < 0.0001. CIA, collagen-induced arthritis; pLN, popliteal lymph node; PCoA, principal coordinates analysis.

We then attempted to identify the metabolite responsible for the prompt pro-resolving effect to continue with targeted mechanistic analyses. Therefore, CIA mice (30 dpi) were orally treated with histamine at concentrations as identified in histamine ELISA of stool extracts from the samples from patients with MS or RA (175.2 nM ± 210.8) (Supplemental Figure 2A). Oral treatment with 125 nM histamine at the peak of disease rapidly improved clinical arthritis scores in CIA mice within 5 days of treatment (Figure 2A). Histological analysis of hind paws confirmed this observation, showing reduced inflammatory lesions in the joints of histamine-treated CIA mice (Figure 2B). Multiplex cytokine and chemokine immunoassays of respective sera specifically revealed differences in CCL5 and CCL2 immune cell chemotactic cytokines, whereas other mediators remained unchanged (Figure 2C and Supplemental Figure 2, B–S). Further, β-diversity analysis together with taxonomic profiling of the bacterial community following 16s rRNA amplicon sequencing identified changed intestinal microbiota compositions after oral histamine treatment, again with an increased relative abundance of *Lactobacillaceae* (37) (Figure 2, D–F). Furthermore, members of this family, such as *Lactobacillus reuteri*, have been described as histamine producers in the intestine. Histamine produced by *Lactobacillus* inhibited proinflammatory cytokine production (38). This led us to investigate whether microbial-secreted histamine would have similar pro-resolving effects as orally supplemented histamine on synovial inflammation. Recently, Barcik et al. (39) developed an *E*. *coli* BL21 strain that was genetically modified to express the *Morganella morganii*–derived HDC (*E*. *coli* HDC^+^), which is responsible for catalyzing the decarboxylation of histidine to histamine. Oral transfer of *E*. *coli* HDC^+^ at the peak of disease significantly reduced arthritis in CIA mice compared with *E*. *coli–*treated or untreated CIA control mice (Figure 2G and Supplemental Figure 2, T–V).

Histamine was shown to exert its effects via 4 histamine receptors, H1R–H4R (40). These receptors differ in their affinity for histamine, with H1R and H2R having a lower affinity and H3R and H4R having a higher affinity (41). Oral treatment of CIA mice with selective H1R–H4R agonists at the peak of disease (30 dpi) identified that only the H3R agonist replicated the strong pro-resolving effects of histamine itself (Figure 2H). This pro-resolving effect was independent from the type of H3R agonist used, as both R(-)-alpha-methylhistamine dihydrochloride (RαMH) (42) and immethridine dihydrobromide (43) showed similar pro-resolving effects (Supplemental Figure 3A). To confirm that only a site-specific increase of histamine in the intestine was initiating the resolution of arthritis, we compared i.p. and intrathecal to oral treatment with the H3R agonist in CIA mice (Supplemental Figure 3, A and B). Only oral delivery showed significant pro-resolving effects on arthritis along with increased intestinal length (Supplemental Figure 3C) as an indicator for reduced intestinal inflammation. Moreover, with increasing H3R agonist concentrations, pro-resolving effects disappeared and even exacerbated clinical arthritis scores (Supplemental Figure 3D). Although t-distributed stochastic neighbor embedding (t-SNE) plots of flow cytometry analysis in the spleen did not show differences in cell clustering with H3R agonist treatment (Figure 2I), CD4^+^ T cells and Th17 cells decreased in the synovial tissue (Figure 2, J and K), and Th17 cells, neutrophils, and CD8^+^ T cells decreased in the draining popliteal lymph node (pLN) after H3R agonist treatment (Figure 2, L–N). As previously shown by others and us, a key mechanistic contributor to the antiinflammatory effect of C3 is the induction of Tregs (44, 30). However, H3R agonist treatment did not induce Tregs (Supplemental Figure 3E). Therefore, the pro-resolving mechanism of C3-induced intestinal histamine is independent of the classical C3-associated Treg induction. Together, these results demonstrated that low-level histamine concentrations in the intestine promote the resolution of arthritis via local H3R signaling.

### H3R agonist stimulates the enteric nervous system in arthritic mice, causing antiinflammatory responses.

**Figure 3:**
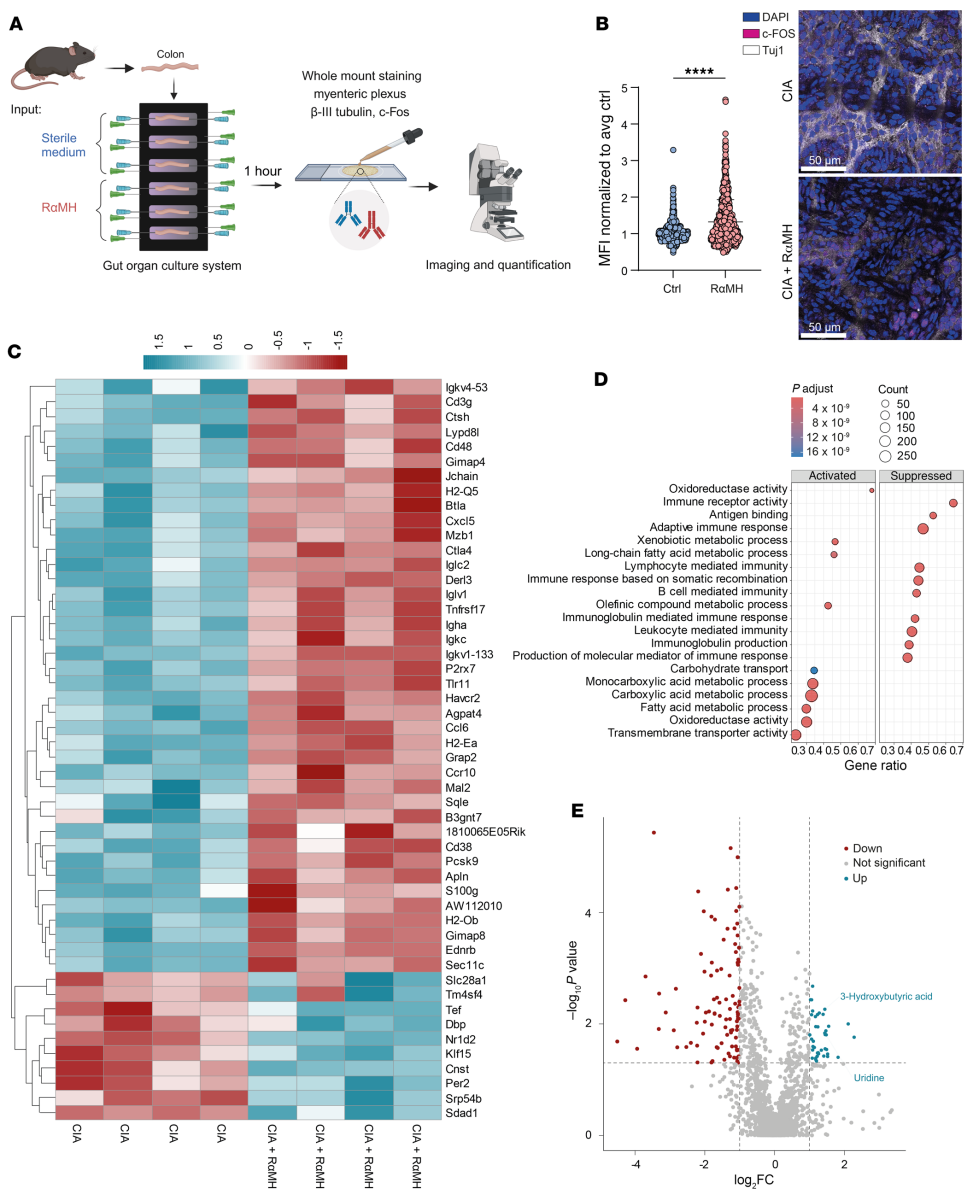
H3R agonist stimulates the ENS in arthritic mice, causing antiinflammatory responses. (**A**) Experimental layout of ex vivo intestinal organ culture system. This overview was generated with BioRender. (**B**) Normalized MFI of c-FOS in βIII-Tubulin^+^ enteric neurons after RαMH stimulation and example images. Scale bars: 50 μm. (**C**) Heatmap of the top 50 regulated genes identified by RNA-Seq of intestinal tissue of CIA mice after in vivo RαMH treatment. (**D**) Gene set enrichment analysis (GSEA) of intestinal RNA-Seq data. (**E**) Volcano plot of untargeted metabolomics data of serum of CIA mice ± RαMH (cutoffs: *P* < 0.05 and –1 < log_2_fc < 1). Data are expressed as mean ± SD. Statistical difference was determined by Student’s *t* test. ****P < 0.0001.

Our results showed that low-dosage, bacteria-derived histamine induced resolution of inflammation via specific H3R activation in the intestinal tract. H3R is mostly expressed on cells of the nervous system. In the intestine specifically, expression is interestingly limited to cells of the enteric nervous system (ENS) (45) and a very low number of endocrine cells (22). To assess H3R-induced changes in the ENS, we applied an ex vivo gut organ culture system (46) that maintains tissue architecture, yet allows tight experimental control, to perform whole-mount staining on the myenteric plexus after H3R agonist stimulation (Figure 3A). The *c-fos* gene is induced by a broad range of stimuli and has been commonly used as a reliable marker for neuronal activity (47). Histological MFI quantification in WT ex vivo gut organ cultures after H3R agonist (RαMH) treatment revealed increased *cFOS* nuclear localization in myenteric neurons positive for βIII-Tubulin (*Tuj1*) (Figure 3B), consistent with previously published results showing increased excitation of enteric neurons through H3R activation (48).

Bulk RNA-Seq experimental data from intestinal tissues of CIA mice treated with H3R agonist (RαMH) revealed significant differences in gene expression profiles over nontreated CIA mice (Figure 3C). Gene ontology analysis showed that oral H3R agonist treatment of CIA mice induced a prominent inflammation-suppressing phenotype in the intestinal tissue (Figure 3D). The most suppressed genes were associated with adaptive immune response (*CD3g*, *Jchain*, *Btla*, *Tnfrsf17*), lymphocyte-mediated immunity (*Iglc2*), and B cell–mediated immunity (*IL7R*, *Igha*) (Supplemental Figure 4). These findings were further supported by untargeted metabolomics analysis of serum metabolites (Figure 3E). Metabolites that were increased upon H3R stimulation were uridine and 3-hydroxybutyric acid, which are not only known for their antiinflammatory properties (49, 50) but also for their role in neuroprotection (51, 52) and suppression of microglia responses (53). Taken together, these data suggest that local H3R activation in the intestine induces an antiinflammatory milieu and increases the neuronal activity of enteric neurons.

### Microglia depletion impairs pro-resolving effects of histamine.

**Figure 4:**
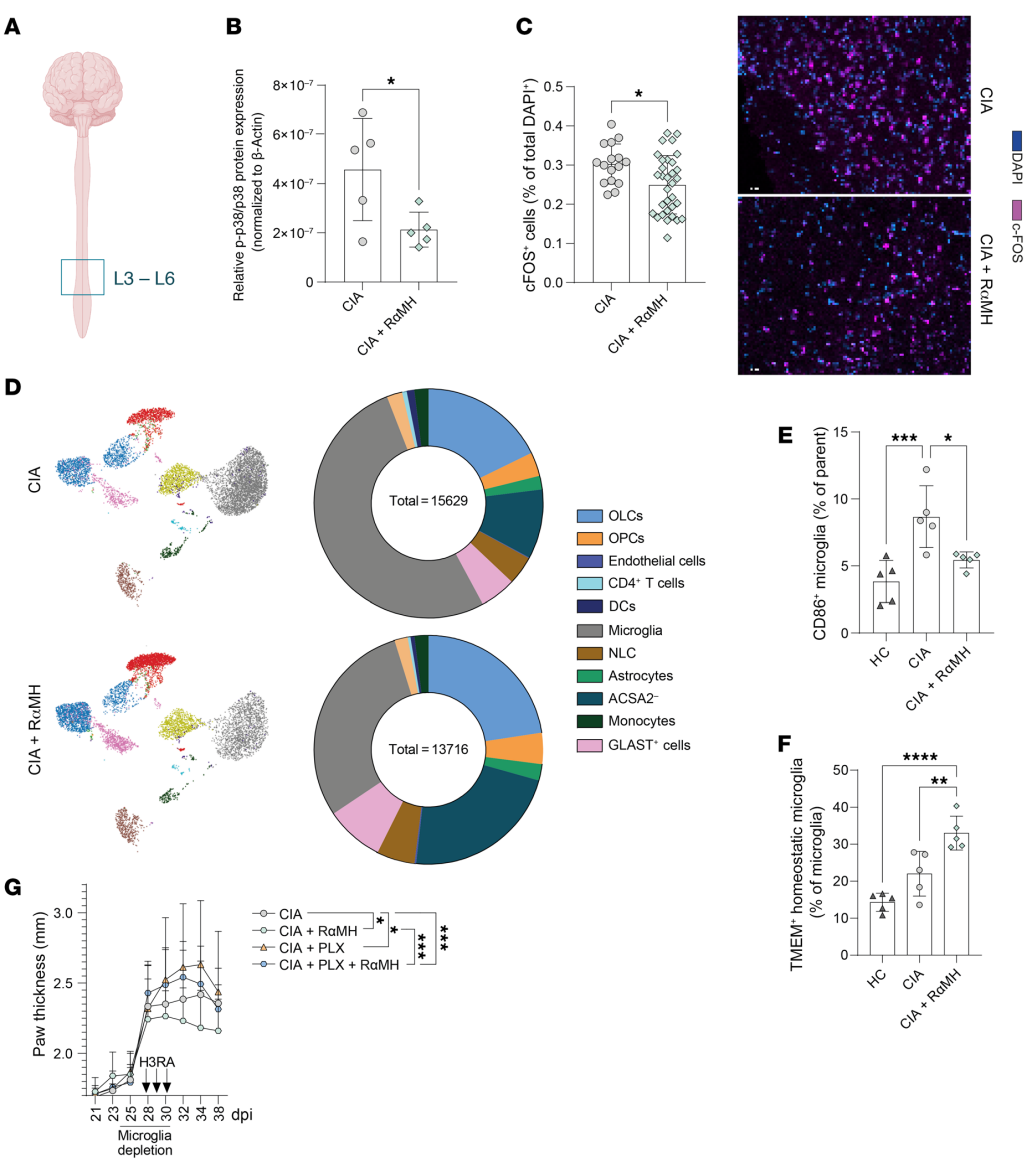
Microglia depletion impairs histamine’s pro-resolving effects. (**A**) Visual representation of the analyzed L3–L6 area of the spinal cord. This overview was generated using BioRender. (**B**) Quantification of phosphorylated p38 protein expression in the L3–L6 area of the spinal cord normalized on β-actin (Western blot). (**C**) Quantification of c-Fos^+^ cells in the spinal cord and example images of immunofluorescence-stained slides. (**D**) UMAP plot of spinal cord cells isolated from CIA ± RαMH and donut chart (% of live cells) of the different cell clusters analyzed by spectral flow cytometry. (**E**) CD86^+^ inflammatory microglia. (**F**) TMEM119^+^ homeostatic microglia. (**G**) Clinical arthritis score shown as paw thickness (mm) of CIA mice with and without microglia depletion (25–30 dpi) ± RαMH at the peak of disease. Data are expressed as mean ± SD. Statistical difference was determined by Student’s *t* test (**B** and **C**), 1-way ANOVA (**E** and **F**), and 1-way ANOVA of AUC (**G**). *P < 0.05, **P < 0.01, ***P < 0.001, ****P < 0.0001.

To formally address the contribution of the CNS in histamine-mediated resolution of arthritis, we next characterized cellular changes in the CNS, followed by respective in vivo cell depletion assays. Peripheral inflammation increases *p38* phosphorylation in neurons and microglia, especially in the dorsal horn in the spinal cord (54, 55). As demonstrated by Boyle et al., local inhibition of p38 phosphorylation in the spinal cord suppresses arthritis (15). Analysis of the lumbar area (L3–L6) of the spinal cord (Figure 4A) that innervates the paws (56) after oral H3R agonist treatment in CIA mice showed reduced *c-fos* and *p38* phosphorylation (Figure 4, B and C). Spectral flow cytometry multiparameter analysis of spinal cord single cells revealed significant cellular changes after H3R agonist treatment in CIA mice (Figure 4D), most prominent within the CD86^+^ microglial cell fraction that was restored to the levels observed in healthy control mice (Figure 4E). Further microglial characterization revealed a shift from inflammatory back to TMEM119^+^ homeostatic microglia (Figure 4F). Interestingly, when we treated microglia, astrocytes, or neurons directly with an H3R agonist in vitro, we did not observe lower levels of inflammatory gene expression or of CCL2 and CCL5 chemokine secretion (Supplemental Figure 5). When applying oral H3R agonist treatment around peak disease in the EAE animal model for MS, where microglial cells were shown to promote inflammation (57, 58), we observed significantly attenuated clinical scores and reduced inflammatory microglial cells in the spinal cord (Supplemental Figure 6), as seen in CIA mice. Next, we investigated the influence of microglia in CIA mice using the colony-stimulating factor 1 receptor inhibitor, PLX5622, to deplete the microglia population (58–60). Effective depletion was confirmed 3 days after the last PLX5622 treatment in CIA mice (Supplemental Figure 6G). After short-term PLX5622 treatment (25–30 dpi) in CIA mice, the prominent H3R agonist–mediated pro-resolving effects were lost when microglia were depleted (Figure 4G). Taken together, these results show that H3R activation in the intestine results in a phenotypic switch from proinflammatory to homeostatic microglial cells in the spinal cord, which are essential for the H3R-induced resolution of arthritis.

### Oral H3R agonist treatment influences microglia function in the spinal cord.

**Figure 5:**
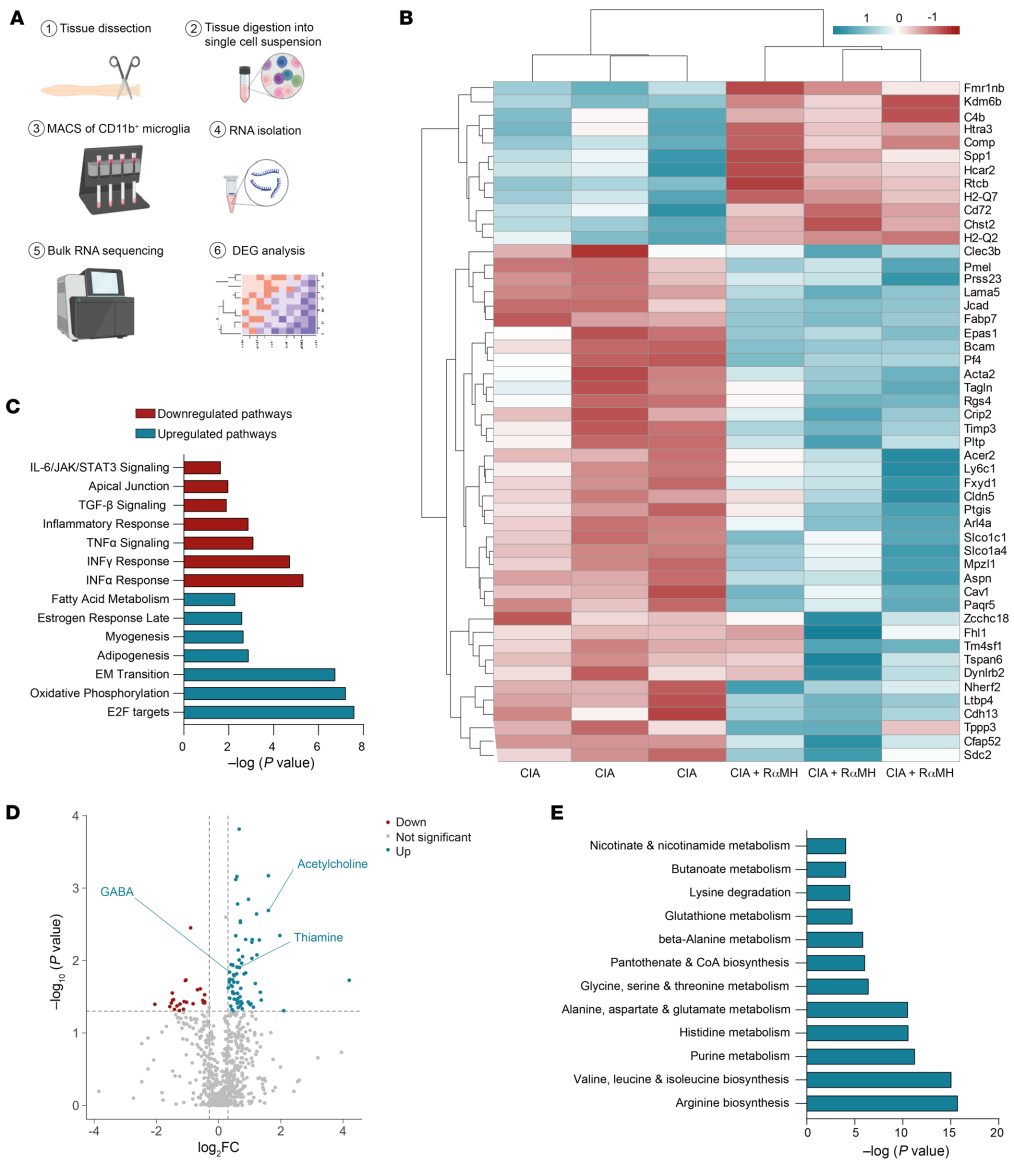
Oral H3R agonist treatment reverses the proinflammatory environment of the CNS. (**A**) RNA-Seq experimental layout (generated using BioRender). (**B**) Heatmap of the top 50 regulated genes in CD11b^+^ cells of the spinal cord in CIA mice ± RαMH. (**C**) Gene set enrichment analysis of top 7 upregulated and downregulated hallmark pathways in the spinal cord. (**D**) Volcano plot of untargeted metabolomics data of spinal cord tissue supernatant of CIA mice ± RαMH (cutoffs were *P* < 0.05 and –0.3 < log_2_fc < 0.3). (**E**) The most upregulated metabolic pathways were identified using MetaboAnalyst.

Our finding that microglia are essential for the intestinal histamine-induced resolution of arthritis prompted us to analyze their transcriptomes in CIA mice after oral H3R agonist treatment. Therefore, CD11b^+^ spinal cord microglia were isolated from CIA mice after oral H3R treatment and processed for bulk RNA-Seq (Figure 5A and Supplemental Figure 6H). Microglia from H3R agonist–treated CIA mice showed a significantly altered gene expression profile, predominantly characterized by an increase of immune modulatory genes such as *Fmr1nb*, *C4b*, *Spp1*, and *CD72* (61–64) (Figure 5B). Gene set enrichment analysis (GSEA) of hallmark genes revealed an overall antiinflammatory phenotype. Inflammatory pathways such as IL-6, JAK/STAT3, and TNF-α signaling were downregulated, whereas antiinflammatory pathways such as oxidative phosphorylation were upregulated (Figure 5C). Further, analysis of the microglial microenvironment by untargeted metabolomics in spinal cords of H3R agonist–treated CIA mice identified a significantly changed secreted metabolite pattern of increased thiamine, acetylcholine, and GABA in lumbar spinal cord tissue supernatants (Figure 5D). Of note, identical spinal cord metabolites were previously linked to attenuated clinical scores in RA (65, 66) by reducing *p38* phosphorylation in the CNS (15, 67). Metabolite set enrichment analysis of identified spinal cord metabolites revealed aspartate and purine metabolism as well as arginine biosynthesis among the highest enriched metabolites (Figure 5E). L-arginine was previously shown to inhibit arthritis and associated inflammatory bone loss in mice (68). In addition, patients with RA exhibit lower purine metabolism activity (69), and a shortage of aspartate was shown to fuel synovial inflammation in RA (70). Together, these data identify oral histamine as a pro-resolving regulator of microglial gene expression and spinal cord metabolites.

### Intestinal H3R signaling reduces vascular leakage in inflamed paws.

**Figure 6:**
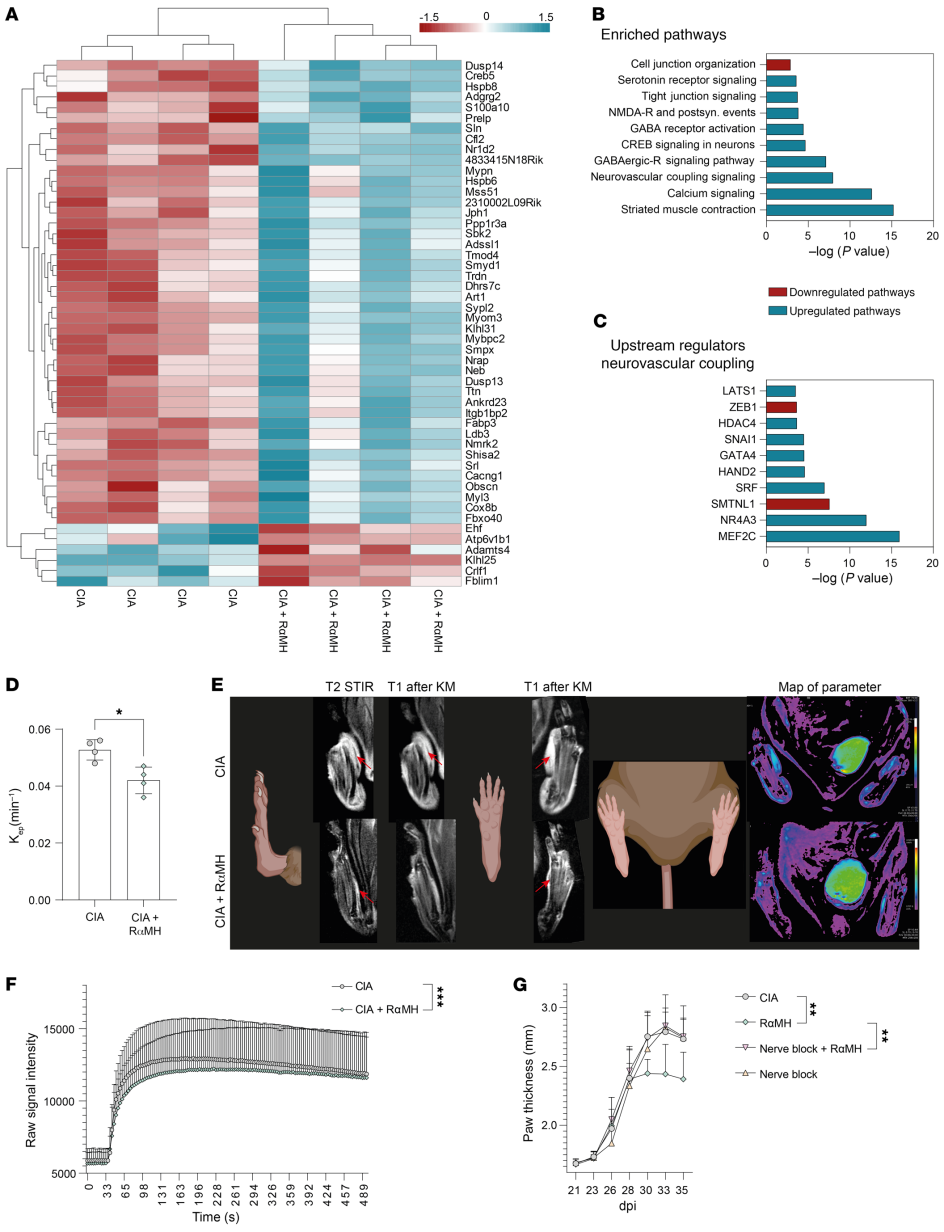
Intestinal H3R signaling reduces vascular leakage in inflamed paws. (**A**) Heatmap of the top 50 regulated genes in CD11b^–^ cells of the nervus plantaris in CIA mice ± RαMH. (**B**) Most enriched pathways identified by IPA (QIAGEN). (**C**) Top 10 upstream regulators of the enriched neurovascular coupling pathway identified by IPA (QIAGEN). (**D**) K_ep_ (transfer constant) in the paws of CIA mice ± RαMH on third treatment day. (**E**) Example MRI of T2 STIR, T1 POST KM, T1 POST KM, and map of parameter of CIA mice ± RαMH. (**F**) Raw signal intensity of DCE measurement over time of CIA mice ± RαMH. (**G**) Clinical arthritis score shown as paw thickness (mm) of CIA mice with or without QX-314 and bupivacaine-induced nerve blockage ± RαMH (*n* = 5) at the peak of disease. Data are expressed as mean ± SD. Statistical difference was determined by Student’s *t* test (**D**), Student’s *t* test of AUC (**F**), and 1-way ANOVA of AUC (**G**). *P < 0.05, **P < 0.01, ***P < 0.001.

Microglia sense neuronal activity and can directly modulate their functions (71). In RA models, sciatic nerve branches were shown to control vascular leakage in arthritic paws (72–74). Therefore, plantar nerve fibers were isolated from the peak of activity of CIA after oral H3R agonist treatment. Sort-purified CD11b^–^ nerve cells were analyzed by bulk RNA-Seq (Figure 6A). The genes most differentially expressed included representatives of several signaling pathways critical for regulating cell and tight junction organization as well as GABA receptor activation and neurovascular coupling, as indicated by enriched pathway analysis (Figure 6B). Further analysis of upstream regulators for neurovascular coupling signaling identified nuclear receptor (NR) *Nr4a3* and *Mef2c* as most significantly upregulated, both described as involved in vascular biology and microglial inflammatory responses (75, 76) (Figure 6C and Supplemental Figure 7, A and B). The smoothelin-like protein 1 (SMTNL1) upstream regulator was most reduced in plantar nerve CD11b^–^ cells, similar to what was found in K/BxN mice after denervation of the sciatic nerve (74) (Figure 6C).

To analyze actual changes in vasoconstriction and vasodilation in CIA paws, in vivo MRI was performed at 28 and 31 dpi, before and after oral H3R agonist treatment, respectively. Reduced K_ep_ values, indicative of reduced vascular leakage, were found in H3R agonist–treated mice (Figure 6D and Supplemental Table 1), along with overall lower raw signal intensity after contrast agent application (Figure 6, E and F). Furthermore, the tendency to lower inflammatory area and paw thickness could be identified by magnetic resonance tomography as soon as the last H3R agonist intervention administration (Supplemental Figure 7, C and D). Also, a reduction of synovial CD4^+^ T cells was visible 8 days after the last RαMH treatment (Supplemental Figure 7E). To investigate the dependence between the microglia changes in the spinal cord and the vascular leakage in the joints, we used a pharmacological approach to temporarily shut down nerve activity (73). QX-314, a membrane-impermeable lidocaine derivative that must enter a cell to block sodium channel conductance, was locally injected in combination with bupivacaine in the footpad minutes before H3R agonist treatment on the peak activity days of CIA. QX-314 treatment did not directly affect paw thickness in CIA mice, but abrogated the antiinflammatory effects after oral H3R agonist administration (Figure 6G). Taken together, these data suggest that local intestinal H3R activation restores vascular leakage in the inflamed joints by affecting centrally controlled local nerve innervation.

### Propionate supplementation increases local histamine levels in patients with RA or MS.

**Figure 7:**
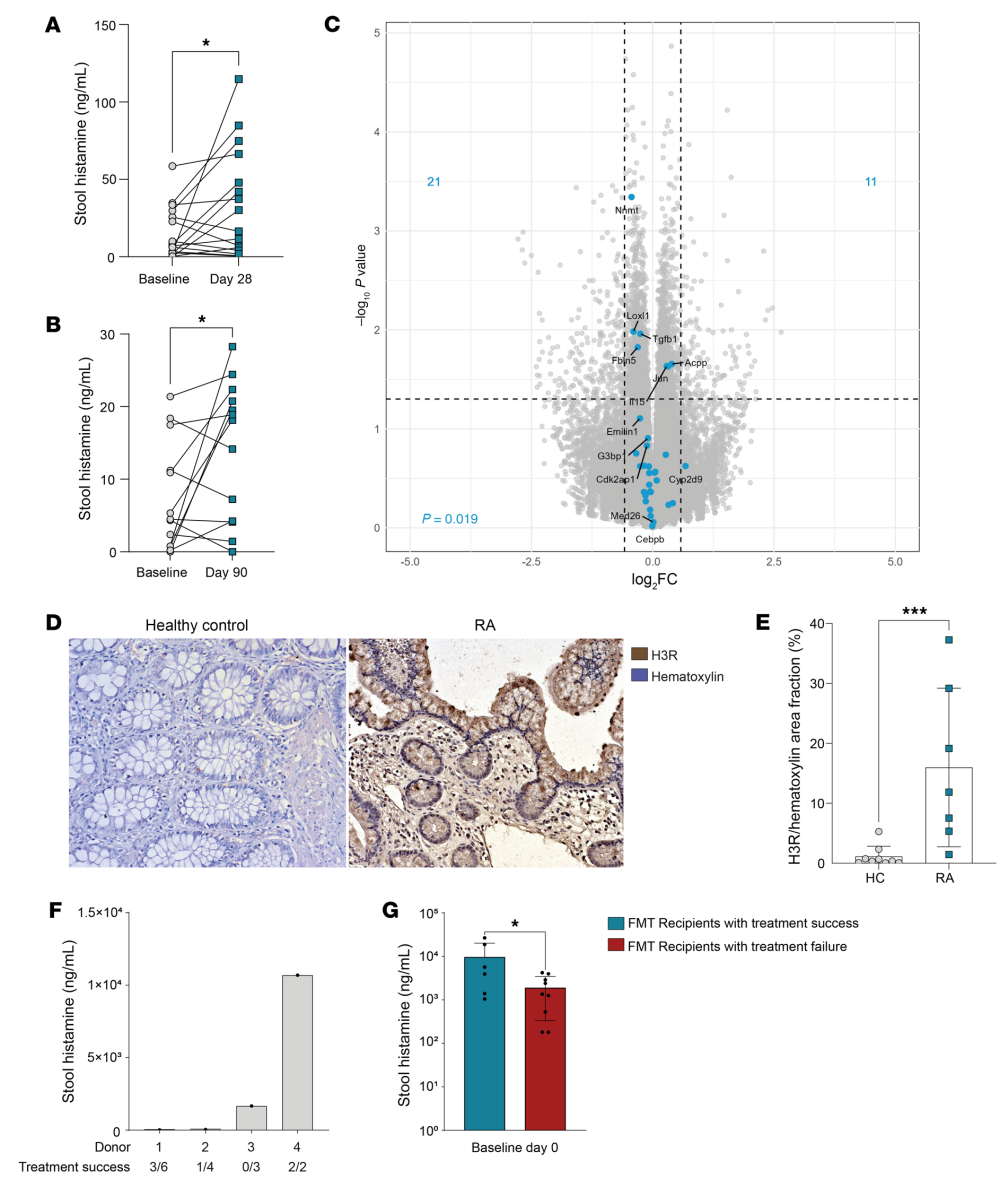
Propionate supplementation increases local histamine levels in patients with RA or MS. (**A**) Histamine levels in stool extracts from patients with RA before and after 28 days of propionate supplementation. (**B**) Histamine levels in stool extracts from patients with MS before and after 90 days of propionate supplementation. (**C**) Volcano plot of RNA-Seq data from ex vivo colon culture infused with stool samples from patients before and after 90 days of propionate supplementation. Overlay with pathway genes of H1R and H2R activation (GSEA pathway: pos histamine response network). (**D**) Example images of histology staining of ileum biopsies from healthy controls or patients with RA stained for H3R. (**E**) Quantification of histological H3R staining of ileum biopsies from patients and healthy controls. (**F**) Histamine levels in stool processed into fecal microbiota transplants (FMTs) from healthy donors (107). FMTs were given to patients with active arthritis despite ongoing treatment with a steady-state dose of methotrexate. After 26 weeks, patients were categorized as being either treatment failures or treatment responders, according to the European psoriatic arthritis recommendations (108). FMT products of donors 1, 2, 3, and 4 were given to 6, 4, 3, and 2 patients, respectively. (**G**) Histamine was measured from the stool of patients with psoriatic arthritis at baseline before FMT. Data are expressed as mean ± SD. Statistical difference was determined by paired *t* test (**A** and **B**) and Student’s *t* test (**E**). *P < 0.05, ***P < 0.001.

To translate our findings to human disease, we utilized samples from 2 human studies, where patients with RA or MS were administered supplementation with the SCFA propionate over the time course of several weeks. In the ProDarMi study (German Clinical Trials Register ID: DRKS00023985), healthy participants were compared with patients with RA, psoriatic arthritis, psoriasis, ankylosing spondylitis, or at risk for RA. The publication of the initial clinical studies showed the beneficial effect of C3 or high-fiber supplementation on disease activity of the patients (77–80). To elucidate whether this effect of propionate was due to an increase in local histamine levels in the gut in the human setting, we analyzed stool samples from these patients at baseline and after 28 days (patients with RA) or 90 days (patients with MS) of C3 treatment. Interestingly, C3 supplementation over the course of 28 and 90 days significantly increased histamine levels in the stool samples of the patients (Figure 7, A and B). To further elucidate the role of microbiota-derived histamine, we reanalyzed RNA-Seq data from the ex vivo gut explant model utilized by Duscha et al. (77), where the gut explants were treated with the microbiota of patients before and after C3 supplementation. Here, we looked at genes that are associated with H1R and H2R activation. Interestingly, H1R- and H2R-associated genes (histamine response network) were downregulated after C3 treatment (Figure 7C), although local histamine levels were significantly increased. This observation supports our hypothesis that this low-level histamine derived from microbiota acts via H3R activation and does not induce allergy or intolerance-associated reactions mediated by H1R or H2R activation. Furthermore, histological analysis of H3R expression in ileal biopsies in patients with RA and healthy controls revealed low levels of H3R expression in the healthy controls, consistent with previously published data (45). In patients with RA, however, H3R expression in ileal tissues was strongly increased (Figure 7, D and E). To further strengthen the translational aspect of our study, we had the unique opportunity to retrospectively analyze FMT samples from the published FLORA trial (ClinicalTrials.gov NCT03058900) (81) within an ongoing collaboration. Our analysis revealed that FMT recipients who received stool samples with high histamine concentrations showed a 100% improvement in clinical response in a small sample group (Figure 7F). Furthermore, we found significant differences in fecal histamine concentrations among FMT recipients, with higher histamine levels associated with clinical improvement (Figure 7G). Taken together, these data indicate that our results in preclinical models may be applicable to human patients.

## Discussion

The concept that the nervous system senses environmental stimuli and transmits these signals to immune cells to maintain tissue homeostasis is well-established (82). However, during chronic arthritic inflammation, it has been shown that the nervous system can exert both proinflammatory and antiinflammatory functions. For example, noninvasive electrical vagus nerve stimulation was shown to be effective in attenuating arthritis in CIA mice and patients with RA (83, 84). On the other hand, denervation of the sciatic nerve protected mice from K/BxN serum transfer arthritis, and individuals with paralysis on one side of the body developed arthritis only on the neurologically unaffected contralateral side (74, 85).

So far, irrespective of the clinical effects, the regulation of arthritis by the CNS has only been investigated in a two-dimensional approach between the CNS and the joints. Here, we identified spinal cord microglia as an essential gut-joint interface responsible for transmitting the antiinflammatory pro-resolving effect of microbiota-derived histamine to the joints, thereby extending the neuroimmunomodulatory concept in RA to a third dimension, the gut.

Building on seminal work from Firestein (15–17) and Straub (86–88), who postulated the CNS as a potential target for the treatment of rheumatic diseases, we experimentally confirmed their previous observations of reduced c-Fos protein expression and p38 phosphorylation locally in the spinal cord after intrathecal application of p38 or TNF-α inhibitors, simply by oral histamine supplementation. That is relevant because peripheral inflammation increases the percentage of neurons with p38 activation in the spinal cords of mice (54).

By involving higher structures of the brain stem, the CNS effectively controls systemic immune responses broadly via the hypothalamic pituitary axis and the release of antiinflammatory glucocorticoids by the adrenal cortex. These effects are in contrast to the finding that somatic sensory afferent fibers in arthritic paws of mice are under local control of the spinal cord and send signals back to the periphery through antidromic action potentials of the dorsal root reflex (89, 90). This reflex is mainly proinflammatory through the recruitment of inflammatory cells and by promoting vasodilation and vessel leakiness, as indicated by a study showing somatic denervation in mice protected against inflammatory arthritis (90). We showed by in vivo MRI that shortly after oral H3R agonist treatment, vessel leakiness in the inflamed paws of CIA mice was likewise reduced, and by temporarily shutting down nerve activity, the pro-resolving effect of histamine was lost.

The autonomic nervous system is the part of the CNS that regulates involuntary body functions and is considered essential for the control of regional homeostasis at the level of individual organs. The autonomic nervous system comprises two main branches: the sympathetic and parasympathetic. Parasympathetic stimulation was shown to suppress peripheral activation via the cholinergic reflex that is triggered by the major parasympathetic neurotransmitter acetylcholine (91, 92). Interestingly, acetylcholine was among the most upregulated metabolites identified in spinal cord tissues after oral H3R agonist treatment in the current study, which is in accordance with previous reports showing H3R-dependent acetylcholine release by histaminergic neurons (93). In preclinical RA models, acetylcholine was shown to effectively attenuate arthritis, and genetic knockdown of the alpha7 nicotinic acetylcholine receptor (α7nAChR) increased CIA severity in mice (17, 66, 94, 95).

Next to acetylcholine, we found that GABA was significantly increased in spinal cord tissues after oral H3R treatment. GABA is a nonproteinogenic amino acid that is widely present in microorganisms, plants, and vertebrates and known to act as an inhibitory neurotransmitter in the CNS. Cadherin-13 was shown to be a critical regulator of GABAergic modulation (96), and we identified it as among the most significantly upregulated genes in sort-purified microglia after oral H3R agonist treatment (Figure 5B). GABA agonist treatment effectively improved clinical signs in a rat model of chronic inflammatory pain (97). The role of GABA signaling in arthritis has also been investigated, and one study (98) showed that the GABAergic system counteracted both the development and progression of RA, potentially by dampening T cell and antigen-presenting cell activity (99). Notwithstanding the fact that GABA levels have been shown to be lower in people with RA compared with healthy individuals (100), the role of the GABAergic system in RA is complex and further research is needed to unravel its functions.

Acute peripheral inflammation was shown to increase glutamate in the spinal cord (101) as seen in the spinal cords of CIA mice after H3R agonist treatment. Glutamate, together with acetylcholine and GABA, has been described as a potent modulator of the microglia phenotype, shifting them to an antiinflammatory homeostatic phenotype (102, 103) that we mimicked with the microglia phenotype after H3R agonist treatment. As shown by 2 independent studies on CIA or *TNF-α* transgenic arthritis mouse models and on postmortem brain tissue from people with RA, microglia per se exhibit an inflammatory transcriptomic phenotype during active disease (104, 105). We identified myocyte-specific enhancer factor 2C (MEF2C) as the strongest induced upstream regulator in sorted nerve cells after H3R agonist treatment, and MEF2C was shown to restrain microglial inflammatory response (75). Pharmacological silencing of primary afferents in arthritic joints prevented microglial activation (73). Depletion of activated microglia in the EAE model delayed disease onset, and microglia-specific deletion of the noncanonical NF-κB–inducing kinase impaired EAE disease progression (57, 58). The increase in homeostatic microglia in the CIA plus RαMH group compared with the CIA group is highly relevant to the resolution of inflammation and represents a key focus of our study. Notably, we also observed this increase in the CIA plus RαMH group relative to control mice, which is an intriguing and potentially novel finding that warrants further investigation. This observation may have important implications for the development of prophylactic strategies aimed at enhancing homeostatic microglia during the early stages of disease, potentially mitigating the expansion of inflammatory microglia. Together with published data, our findings suggest a central role for microglia in the gut-specific H3R signaling–mediated resolution of inflammation.

A limitation of this study is that the main conclusions regarding the central role of microglia are based on microglial elimination using PLX5622. Our data showed that in CIA mice, short-term PLX5622 treatment led to the loss of the pronounced pro-resolving effects mediated by the H3R agonist. We used PLX5622 to assess the dependence of these effects on microglia, assuming that its action was restricted to microglia. However, recent evidence suggests that PLX5622 also affects myeloid compartments in the bone marrow and spleen (106). It is important to note that the treatment regimens in this study differ fundamentally from our approach in terms of duration and intensity. Future studies should employ microglia-specific genetic models to refine our findings. Currently, a major limitation of existing genetic microglia depletion models for our study is twofold: first, their often low specificity, and second, the C57BL/6 genetic background of these strains, which renders them resistant to the CIA model used in our study. In addition, it would be helpful if the number of cells per milligram of tissue were also given in the following investigations in order to be able to better estimate the total number of infiltrating cells in the synovium or in the lymph nodes or to determine whether a lower percentage of certain cell subsets is present. Furthermore, our dose-dependent results indicate that the loss of pro-resolving effects and the worsening of arthritis scores at higher H3R agonist concentrations suggest that establishing an effective dosage may be challenging when translating these findings to human disease.

We propose that microbiota-derived histamine in the gut stimulates the enteric nervous system via H3R signaling (48). This tissue-specific response to histamine triggered the restoration of antiinflammatory homeostatic microglia in the spinal cord, where proinflammatory microglia were found to be enriched in preclinical arthritic models and in patients with RA (104, 105). Both the transcription profiles of the microglia and the secreted metabolites in the spinal cord create a local antiinflammatory environment that causes a reduction in vasodilation and an egress of inflammatory cells from arthritic paws via efferent nerve fibers. We also found that disrupting this gut/CNS/joint axis either by depleting microglia in the spinal cord or by blocking nerve fibers in the paws abolished the pro-resolving effect of histamine. In addition, a high-fiber diet or direct SCFA-propionate supplementation increased local histamine concentrations in the gut of patients with RA or MS, thereby supporting resolution of inflammation.

## Methods

### Sex as a biological variable.

For all experiments, female mice were used because of their higher susceptibility to CIA. This aligns with human RA, which is more prevalent in women.

### Mice.

Five- to 6-week-old WT C57BL/6N (Charles River Laboratories) and DBA/1J mice (Janvier) were acclimated for 1 week, followed by a 3-week cohousing period before starting the experiments. All mice were maintained under specific pathogen–free conditions at the Präklinisches Experimentelles Tierzentrum (PETZ) in Erlangen, Germany. Sodium propionate (Sigma-Aldrich) was added to drinking water at a final concentration of 150 mM and changed every 3 days. The animals received water with or without C3 and standard chow (Ssniff Spezialdiäten GmbH) ad libitum.

### Human studies.

The demographic characteristics of the RA C3 supplementation study were as follows: age, 49.46 ± 14.88 years (mean ± SD); females, *N* (%) = 12 (85.71%). The disease-specific characteristics were as follows: disease activity score (DAS) of 28 erythrocyte sedimentation rate, units = 3.1 ± 1.48 (mean ± SD); DAS of 28 C-reactive protein, units = 2.71 ± 1.49 (mean ± SD). Demographic characteristics of the MS study are as follows: age, 51.32 ± 13 (mean ± SD); females, *N* (%) = 12 (42.86%). For the FLORA trial, FMT donors were between 25 and 55 years old. The demographic characteristics of FMT patients were as follows: age, 48.9 ± 16.1 years (mean ± SD); females, *N* (%) = 8 (53%).

### CIA.

CIA was induced in 8-week-old female DBA/1J mice by s.c. injection at the base of the tail with 100 μL of 0.25 mg chicken type II collagen (CII; Chondrex) in CFA (Difco Laboratory) containing 5 mg/mL killed *Mycobacterium tuberculosis* (H37Ra). Mice were rechallenged after 21 days intradermal immunization in the base of the tail with 100 μL of 0.25 mg chicken type II collagen (CII; Chondrex) in incomplete Freund adjuvant. The paws were evaluated for joint swelling 3 times per week. Each paw was individually measured for paw thickness using a caliper or by giving an eye score using a 4-point scale: 0, normal paw; 1, minimal swelling or redness; 2, redness and swelling involving the entire forepaw; 3, redness and swelling involving the entire limb; 4, joint deformity or ankylosis or both. Mean values of paw thickness and summed eye scores are displayed.

### Supplementation of sodium propionate in drinking water.

Mice were supplemented with 150 mM sodium propionate (C3, Sigma-Aldrich) in their drinking water. Drinking water was changed every 2 days to ensure consistent concentration and quality of the solution. Naive mice were treated for 3 weeks as FMT donors, and CIA mice were treated starting from the peak of disease severity (30 dpi).

### FMT.

Donor mice were treated for at least 3 weeks with 150 mM sodium propionate (C3) in the drinking water. For transfer into 5 recipient mice, 2 donor mice were euthanized, and their cecum contents were mixed with 2.5 mL 1 × PBS (for transfer) or H_2_O for fast protein liquid chromatography (FPLC) or untargeted metabolomics. The mixture was filtered through a 100 μm cell strainer. Then, 250 μL of the filtered solution was transferred into the donor mice by oral gavage. To guarantee the stability of the transfer, the procedure was repeated after 2 days. To assess the effect of soluble components or bacterial and cellular matter on ongoing CIA in mice, the stool mixture was centrifuged at 3000*g*. Supernatant fraction was aspirated and pellet was resuspended in PBS before transfer into mice with CIA by oral gavage.

### Culture and transfer of E. coli strains.

Frozen *E*. *coli* glycerol stocks supplied in-house were inoculated in 5 mL Luria and Bertani medium (Roth). Bacteria were incubated overnight in a bacteria shaker at 37°C and 300 rpm. The next day, the OD 450 nm was measured every hour until reaching the exponential phase. DBA1/J mice were treated with *E*. *coli* B21 ± HDC by oral gavage. Each mouse received 1 × 10^8^ per 250 μL dose reconstituted in PBS. Control mice received 250 μL PBS by oral gavage. Treatment was performed twice at days 30 and 32 dpi.

### Fraction-generation by FPLC.

Supernatant was further separated into 5 different size-specific fractions. First, the sample was centrifuged at 100,000*g* for 60 minutes. Supernatant was transferred on a VivaSpin 6 Column with a cutoff of 3 kDa and centrifuged for about 40–60 minutes until the remaining supernatant was 250 μL. Flow-through was collected as fraction 1 for further transfer. Remaining supernatant was collected for separation into 4 further fractions by FPLC in cooperation with Xiang Wei from the Molecular Neurology Department of the Universitätsklinikum Erlangen. Fractions were aliquoted and stored at –80°C until transfer into mice. Supernatant fractions were thawed shortly before transfer into mice at the peak of CIA by oral gavage. Mice were treated for 5 days with 250 μL of the corresponding fraction or PBS as a control.

### Oral treatment with histamine and histamine-receptor agonists.

Histamine and histamine receptor agonists (see Table 1) were dissolved in PBS to a stock concentration of 0.5 mM and stored at –20°C. Histamine treatment concentrations were carefully selected based on histamine concentrations in the stool of patients with RA or MS after C3 treatment. Mice at the peak of CIA were treated with 250 μL histamine (final concentration of 125 nM), H1R agonist, H2R agonist, H4R agonist (900 nM; equipotent to histamine), or H3R agonist (60 nM; 15 times more potent than histamine) by oral gavage for 3 days.

### Microglia depletion.

Microglia were depleted using the CSF1R antagonist PLX5622. Mice received daily i.p. injections with 50 mg/kg PLX5622 (reconstituted in DMSO) or DMSO as control over 5 days.

### Blockage of primary afferents in the paws.

The membrane-impenetrable lidocaine sodium channel blocker QX-314 (2%, Sigma-Aldrich, 552233) was coadministered daily with bupivacaine (10 μg, Sigma-Aldrich, B5274) via intraplantar injection in the hind paws for 3 days in combination with oral H3R agonist treatment in CIA mice at the peak of disease (28–30 dpi).

### EAE.

EAE was induced in 8–12-week-old female C57Bl/6J mice using 150 μg of MOG35–55 (Genemed Synthesis, 110582) mixed with freshly prepared CFA, using 20 mL Incomplete Freund’s Adjuvant (BD Biosciences, BD263910) mixed with 100 mg *Mycobacterium* *tuberculosis* H37Ra (BD Biosciences, 231141) at a ratio of 1:1 (v/v at a concentration of 5 mg/mL). All mice received 2 s.c. injections of 100 μL each of the MOG35-55/CFA mix. All mice then received a single i.p. injection of 200 ng pertussis toxin (List Biological Laboratories, 180) in 200 μL of PBS. Mice received a second pertussis toxin injection at the same concentration 2 days after EAE induction. Mice were monitored and scored daily thereafter. EAE clinical scores were defined as follows: 0, no signs; 1, fully limp tail; 2, hindlimb weakness; 3, hindlimb paralysis; 4, forelimb paralysis; 5, moribund.

### Statistics.

Statistical analyses were performed using GraphPad Prism 9 software. Comparisons between 2 groups were performed using unpaired or paired, 2-tailed Student’s *t* tests. Comparisons between more than 2 groups were performed using 1-way ANOVA and Tukey’s or Dunnett’s multiple-comparison test. *P* values less than 0.05 were considered significant. Details on the statistical analyses are listed in the figure legends.

### Study approval.

The RA C3 supplementation study was approved by the ethics committee of the Department of Medicine at the Friedrich-Alexander University Erlangen-Nürnberg (431_20 B). The study protocol and trial registration can be found in the German Clinical Trials Register (ID: DRKS00023985).

The MS study was approved by the ethics committee of the Department of Medicine at the Ruhr-University Bochum (registration 15-5351, 4493-12, 17-6235), and all study information has previously been published (77).

All experiments were approved by the local ethics authorities of the Regierung of Unterfranken, Germany (55.2-2532-2-424, 55.2-2532-2-1674, 55.2.2-2532-2-1180).

### Data availability.

All relevant data are available from the authors upon reasonable request. The source data underlying Figures 1–7 and Supplemental Figures 1–7 are provided as a source data file. The 16s rRNA-Seq data are deposited in a publicly accessible database, Figshare (https://figshare.com/s/b5a827f0fb182a57b242). The supporting data values are available in the Supporting Data Values file.

## Author contributions

GS, VR, and MMZ designed the project, interpreted results, and wrote the manuscript. KD and ML performed most of the work, analyzed data, edited the manuscript, and made figure panels. LE, ES, HD, MHS, LL, MF, VA, FS, and SL acquired data and provided help with multiple experiments. LA, TRL, and TS performed 16s rRNA-Seq and analyzed 16s rRNA data. HBM, YR, HR, and NY performed and analyzed gut explant experiments. AG and RVT performed and analyzed untargeted metabolomics experiments. JH performed and analyzed the SCFA measurements. DM and FC performed and analyzed human H3R staining. FB and RB performed spinal cord histology. MSK and TE performed the FLORA study and provided the FMT samples. WX performed size exclusion chromatography. AH and CAA provided human MS stool samples and HDC-competent *E*. *coli* strains, respectively. TB performed and analyzed magnetic resonance tomography measurements. TB and KS wrote the animal license approvals. MMZ supervised the project and provided funding. All authors revised and approved the manuscript.

## Supplementary Material

Supplemental data

Supporting data values

## Figures and Tables

**Table 1:**
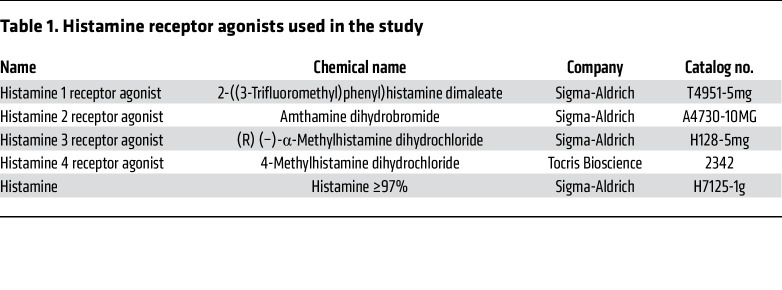
Histamine receptor agonists used in the study

## Version 1 07/10/2025

In-Press Preview

## Version 2 09/16/2025

Electronic publication
