# Supplementary material for: Gut-specific histamine 3 receptor signaling orchestrates microglia-dependent resolution of peripheral inflammation
Source: J Clin Invest. 2025 Jul 10;135(18):e184697. doi: 10.1172/JCI184697 (PMC12435854; doi:10.1172/JCI184697)
Supplement: Supplemental data [file jci-135-184697-s082.pdf]

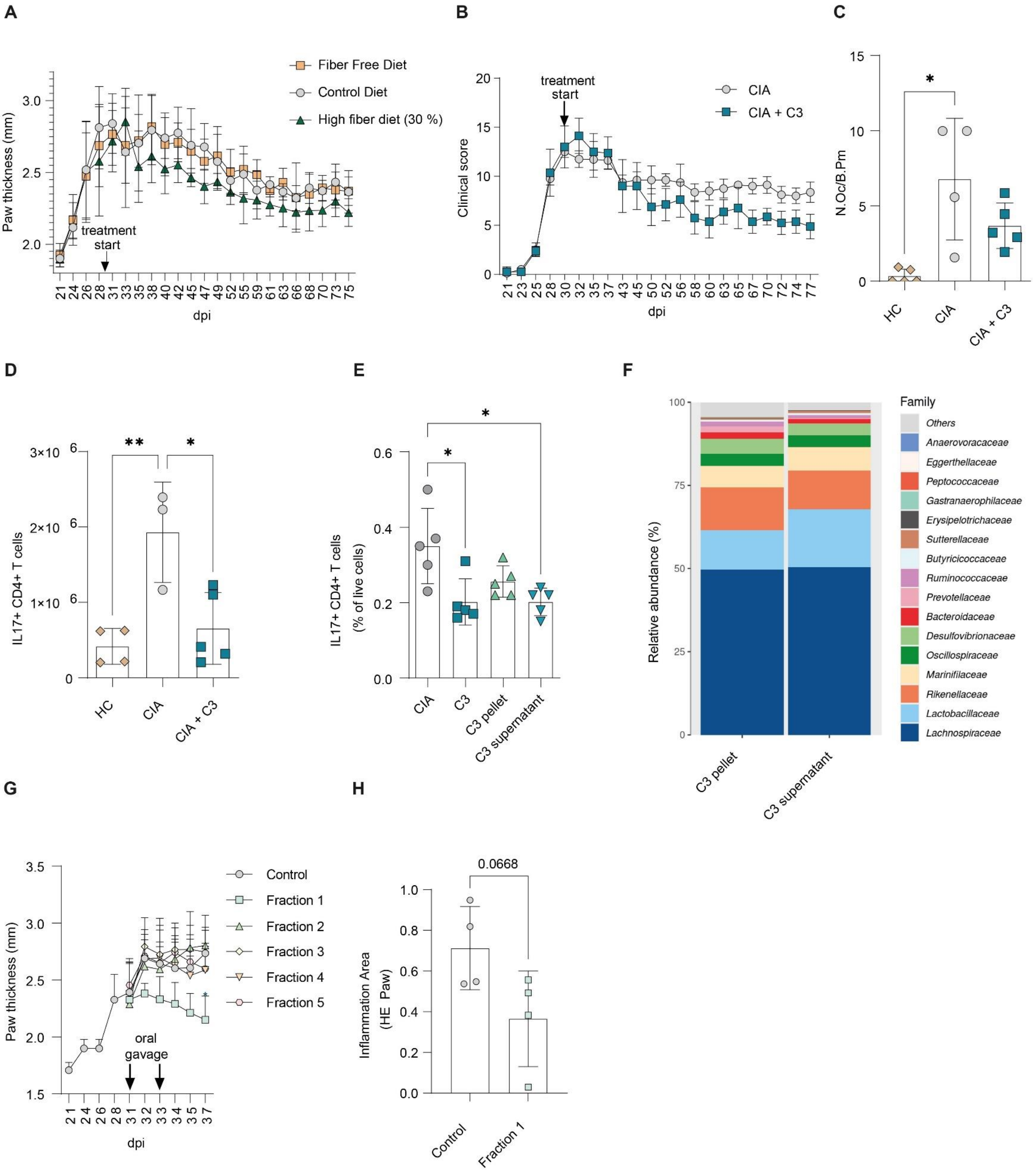
A
Fiber Free Diet
Control Diet
High fiber diet (30 %)
Paw thickness (mm)
treatment start
dpi
B
treatment start
CIA
CIA + C3
Clinical score
dpi
C
N.Oc/B.Pm
HC
CIA
CIA + C3
D
IL17+ CD4+ T cells
3×10 6
2×10 6
1×10 6
HC
CIA
CIA + C3
E
IL17+ CD4+ T cells
(% of live cells)
CIA
C3
C3 pellet
C3 supernatant
F
Relative abundance (%)
Family
Others
Anaerovoracaceae
Eggerthellaceae
Peptococcaceae
Gastranaerophilaceae
Erysipelotrichaceae
Sutterellaceae
Butyricicoccaceae
Ruminococcaceae
Prevotellaceae
Bacteroidaceae
Desulfovibrionaceae
Oscillospiraceae
Marinifilaceae
Rikenellaceae
Lactobacillaceae
Lachnospiraceae
C3 pellet
C3 supernatant
G
Control
Fraction 1
Fraction 2
Fraction 3
Fraction 4
Fraction 5
Paw thickness (mm)
oral gavage
dpi
H
0.0668
Inflammation Area
(HE Paw)
Control
Fraction 1

Supplemental Figure 1. (A) Clinical arthritis score shown as paw thickness (mm) of CIA mice on control diet, a high fiber diet (30 % fiber) or a fiber free diet starting 30 dpi. **(B)** Clinical arthritis score shown as eye score of CIA mice ± 150 mM C3 (n=4-5) in the drinking water starting 30 dpi. **(C)** Number of Osteoclasts (N.Oc) per bone Perimeter (B.Pm) in the hind paw of healthy and CIA mice ± C3 **(D)** Flow cytometric analysis of IL-17+ CD4+ T cells in the spleen of healthy mice and CIA mice ± C3 shown as the total cell numbers. **(E)** IL-17+ CD4+ T cells in the spleen of CIA mice treated with FMT of naïve donors, C3-treated donors, supernatant of C3-treated donors or pellet of C3-treated donors. **(F)** Relative abundance of the bacterial families identified by 16S rRNA sequencing. DNA was collected from the cecum content of CIA mice 5 days after last treatment with FMT of supernatant or pellet of C3-treated donors (n = 4-5). **(G)** Clinical arthritis score shown as paw thickness (mm) of CIA mice receiving different parts of the FMT supernatant separated by size exclusion chromatography by oral gavage. **(H)** Inflammation area in the paws of CIA mice ± Fraction 1. Data are expressed as the mean ± sd. Statistical difference was determined by Kruskal-Wallis test (C) One-way ANOVA (D and E) and t-test (H). $*p < 0.05$; dpi = days post immunization, FMT = fecal microbiota transfer.

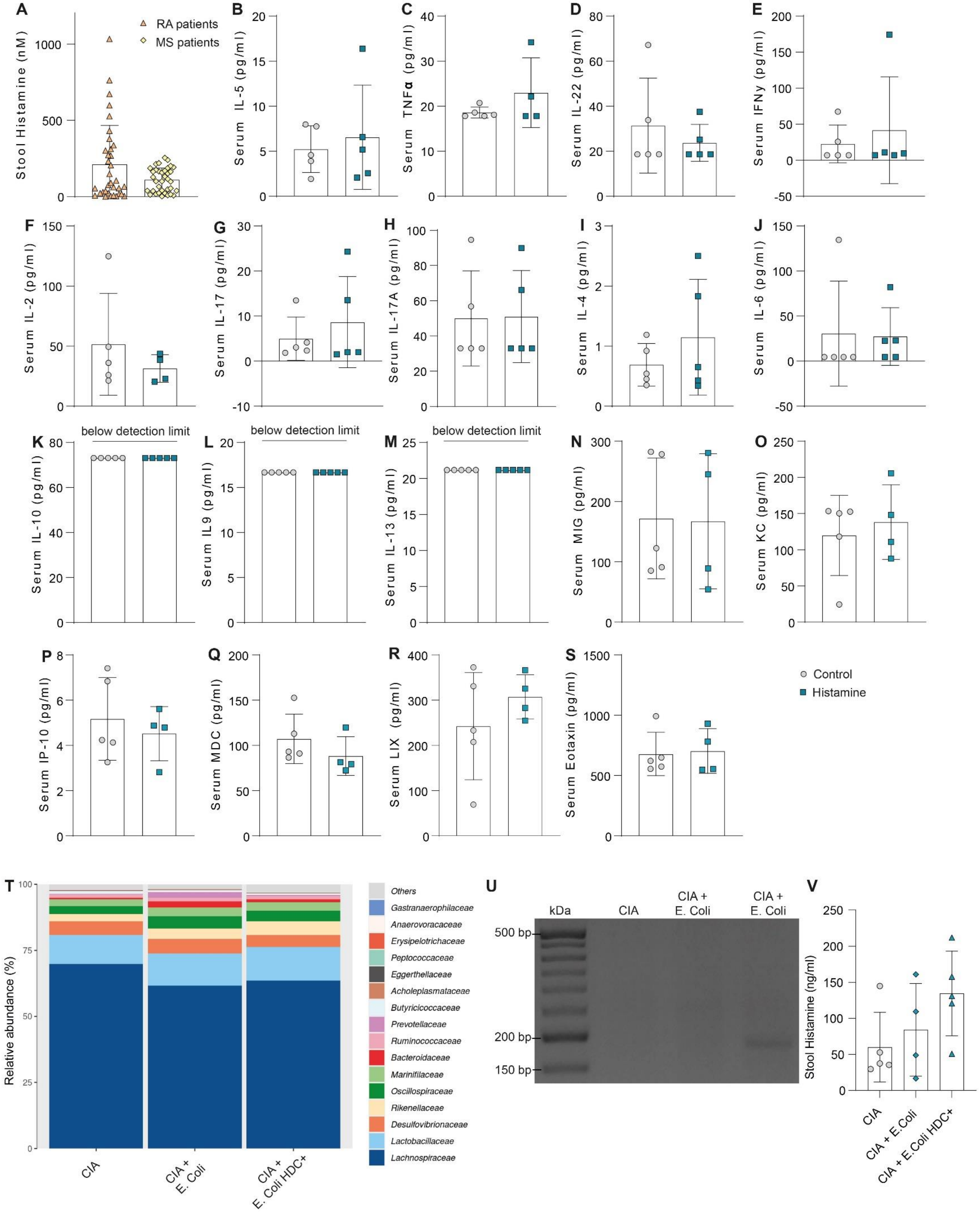
A
B
C
D
E
F
G
H
I
J
K
L
M
N
O
P
Q
R
S
T
U
V
RA patients
MS patients
Stool Histamine (nM)
Serum IL-5 (pg/ml)
Serum TNFα (pg/ml)
Serum IL-22 (pg/ml)
Serum IFNγ (pg/ml)
Serum IL-2 (pg/ml)
Serum IL-17 (pg/ml)
Serum IL-17A (pg/ml)
Serum IL-4 (pg/ml)
Serum IL-6 (pg/ml)
below detection limit
Serum IL-10 (pg/ml)
Serum IL9 (pg/ml)
Serum IL-13 (pg/ml)
Serum MIG (pg/ml)
Serum KC (pg/ml)
Serum IP-10 (pg/ml)
Serum MDC (pg/ml)
Serum LIX (pg/ml)
Serum Eotaxin (pg/ml)
Control
Histamine
Relative abundance (%)
CIA
CIA + E. Coli
CIA + E. Coli HDC+
Others
Gastranaerophilaceae
Anaerovoracaceae
Erysipelotrichaceae
Peptococcaceae
Eggerthellaceae
Acholeplasmataceae
Butyricicoccaceae
Prevotellaceae
Ruminococcaceae
Bacteroidaceae
Marinifilaceae
Oscillospiraceae
Rikenellaceae
Desulfovibrionaceae
Lactobacillaceae
Lachnospiraceae
kDa
500 bp
200 bp
150 bp
Stool Histamine (ng/ml)
CIA + E.Coli
CIA + E.Coli HDC+

Supplemental Figure 2. (A) Stool histamine levels (nM) of RA and MS patients. **(B)** Serum IL-5 **(C)** TNFα **(D)** IL-22 **(E)** IFNγ **(F)** IL-2 **(G)** IL-17 **(H)** IL-17A **(I)** IL-4 **(J)** IL-6 **(K)** IL-10 **(L)** IL-9 **(M)** IL-13 **(N)** MIG **(O)** KC **(P)** IP-10 **(Q)** MDC **(R)** LIX **(S)** Eotaxin. **(T)** Relative abundance of the bacterial families identified by 16S rRNA sequencing. DNA was collected from the cecum content of CIA mice 8 days after last transfer of PBS (Control), *E. Coli* or HDC+ *E. Coli* (n = 4-5). **(U)** PCR amplified E. coli DNA from the cecum of CIA mice 8 days after last transfer of PBS (Control), *E. Coli* or HDC+ *E. Coli* (n = 4-5). **(V)** Histamine levels in the cecum contents of CIA mice 8 days after last transfer of PBS (Control), *E. Coli* or HDC+ *E. Coli*. Data are expressed as the mean ± sd. Statistical difference was determined by Student's t-test. *p < 0.05;

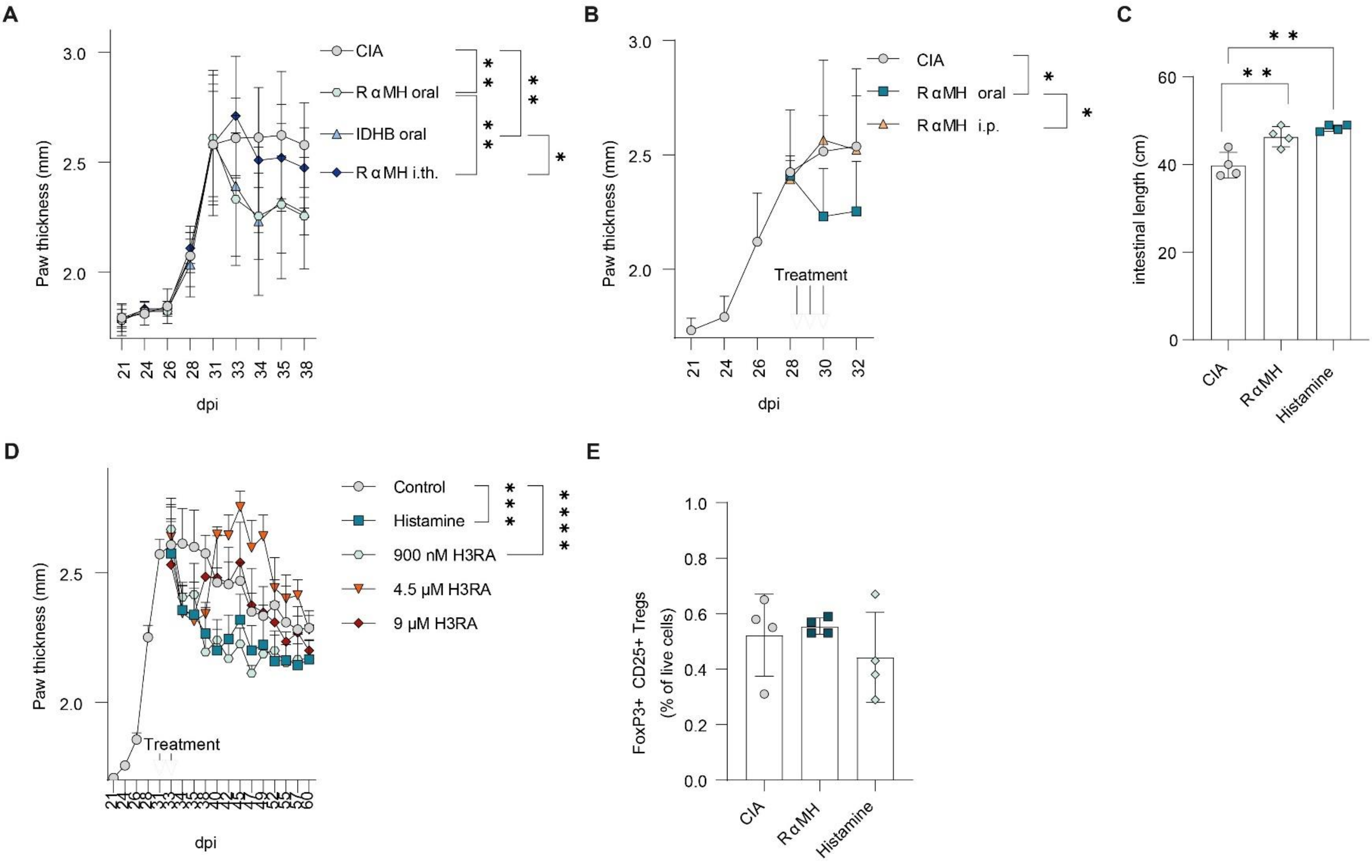

Supplemental Figure 3. (A) Clinical arthritis score shown as paw thickness (mm) of CIA mice treated orally or intrathecally (i.th.) with the H3R agonist RαMH, or orally with the H3R agonist Immethidrine dihydrobromid (IDHB). **(B)** Clinical arthritis score shown as paw thickness (mm) of CIA mice treated orally or intraperitoneally with RαMH. **(C)** Intestinal length of CIA mice ± oral treatment with RαMH or histamine. **(D)** Clinical arthritis score shown as paw thickness (mm) of CIA mice treated orally with histamine or different concentrations of the H3R agonist RαMH. **(E)** FoxP3+ CD25+ regulatory T cells in the spleen of CIA mice ± oral treatment with RαMH or histamine. Data are expressed as the mean ± sd. Statistical difference was determined by One way-ANOVA (C,E) of area under the curve (A,B,D). *$p < 0.05$; **$p < 0.01$; ***$p < 0.001$. dpi = days post immunization.

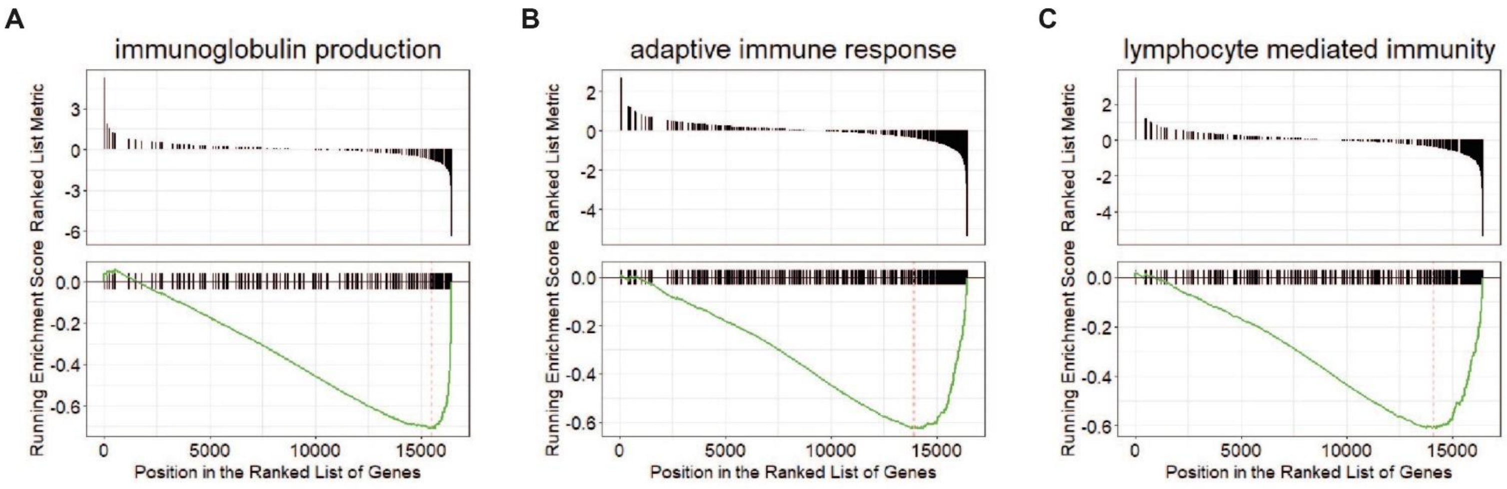

Supplemental Figure 4. (A) Running Enrichment Score and Ranked List Metric of the Immunoglobulin production pathway gene set. **(B)** Running Enrichment Score and Ranked List Metric of the adaptive immune response pathway gene set. **(C)** Running Enrichment Score and Ranked List Metric of the lymphocyte mediated immunity pathway gene set.

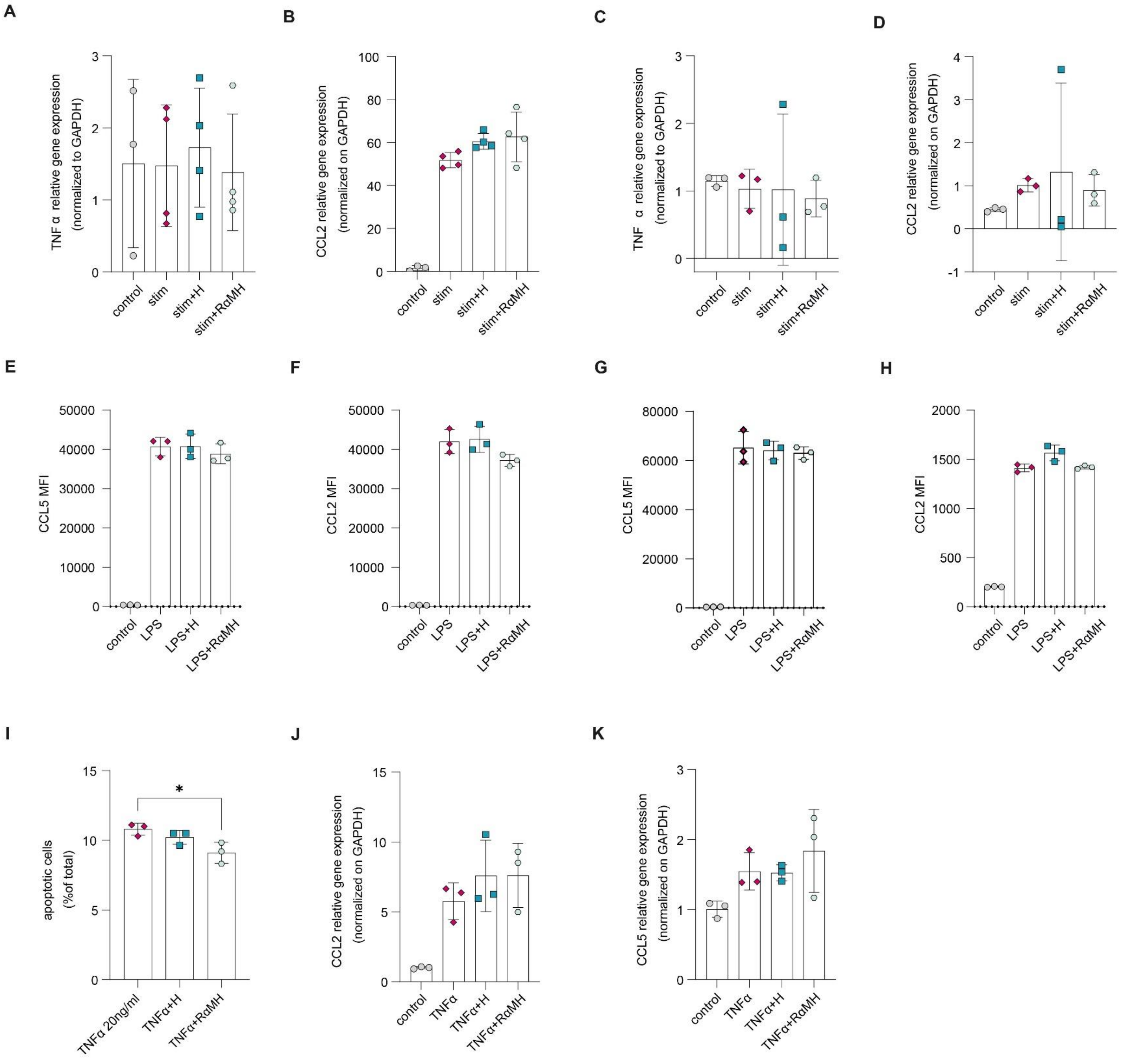

A
TNF α relative gene expression (normalized to GAPDH)
control
stim
stim+H
stim+RαMH
B
CCL2 relative gene expression (normalized on GAPDH)
C
TNF α relative gene expression (normalized to GAPDH)
D
CCL2 relative gene expression (normalized on GAPDH)
E
CCL5 MFI
LPS
LPS+H
LPS+RαMH
F
CCL2 MFI
G
CCL5 MFI
H
CCL2 MFI
I
apoptotic cells (%of total)
*
TNFα 20ng/ml
TNFα+H
TNFα+RαMH
J
CCL2 relative gene expression (normalized on GAPDH)
TNFα
K
CCL5 relative gene expression (normalized on GAPDH)

Supplemental Figure 5. (A) Relative TNFα gene expression of astrocytes ± histamine or RαMH. **(B)** Relative CCL2 gene expression of astrocytes ± histamine or RαMH. **(C)** Relative TNFα gene expression of microglia ± histamine or RαMH. **(D)** Relative CCL2 gene expression of microglia ± histamine or RαMH. **(E)** CCL5 MFI in cell culture supernatant of astrocytes after stimulation with histamine or RαMH. **(F)** CCL2 MFI in cell culture supernatant of astrocytes after stimulation with histamine or RαMH. **(G)** CCL5 MFI in cell culture supernatant of microglia after stimulation with histamine or RαMH. **(H)** CCL2 MFI in cell culture supernatant of microglia after stimulation with histamine or RαMH. **(I)** Apoptotic cells after stimulation of N2A neuronal cell line with TNFα, TNFα + histamine or TNFα + RαMH. **(J)** CCL2 gene expression of N2A neuronal cell line with TNFα, TNFα + histamine or TNFα + RαMH. **(K)** CCL5 gene expression of N2A neuronal cell line with TNFα, TNFα + histamine or TNFα + RαMH. Data are expressed as the mean ± sd. Statistical difference was determined by One way-ANOVA. *p < 0.05; **p < 0.01; ***p < 0.001. stim = stimulated; H = Histamine.

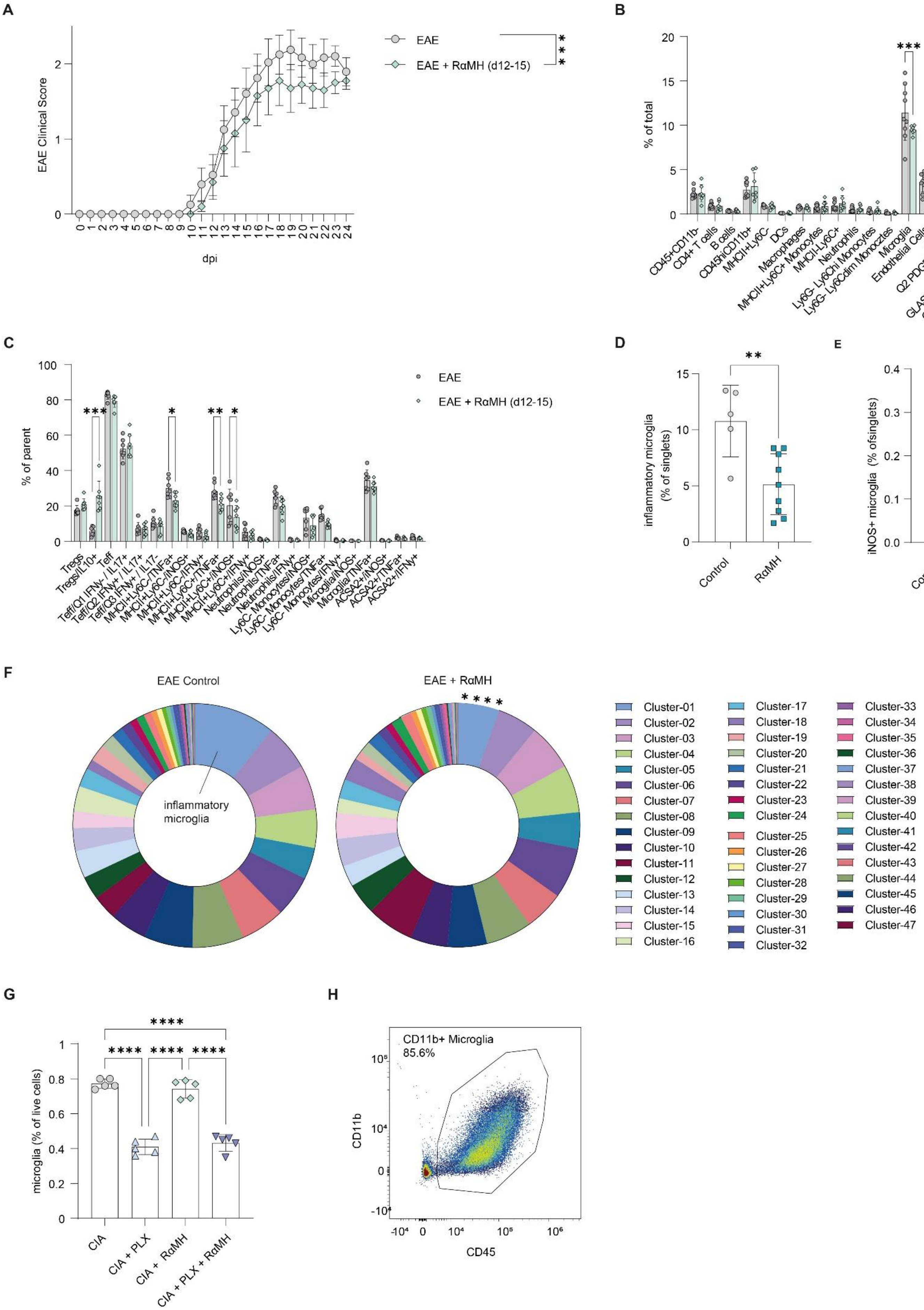
A
EAE Clinical Score
dpi
EAE
EAE + RαMH (d12-15)
***
B
% of total
***
CD45+CD11b-
CD4+ T cells
B cells
CD45hiCD11b+
MHCII+Ly6C-
DCs
Macrophages
MHCII+Ly6C+ Monocytes
MHCII-Ly6C+
Neutrophils
Ly6G- Ly6Chi Monocytes
Ly6G- Ly6Cdim Monocytes
Microglia
Endothelial Cells
OLCs
Q2 PDGFRa+ / O4+
OPCs
GLAST- ACSA2+ astrocytes
GLAST+ ACSA2+ astrocytes
Q3 GLAST+ / ACSA2-
C
% of parent
EAE
EAE + RαMH (d12-15)
Tregs
Tregs/IL10+
Teff
Teff/Q1 IFNγ- / IL17+
Teff/Q2 IFNγ+ / IL17+
Teff/Q3 IFNγ+ / IL17-
MHCII+Ly6C-/TNFa+
MHCII+Ly6C-/iNOS+
MHCII+Ly6C-/IFNγ+
MHCII-Ly6C+/TNFa+
MHCII-Ly6C+/iNOS+
MHCII-Ly6C+/IFNγ+
Neutrophils/iNOS+
Neutrophils/TNFa+
Neutrophils/IFNγ+
Ly6C- Monocytes/iNOS+
Ly6C- Monocytes/TNFa+
Ly6C- Monocytes/IFNγ+
Microglia/iNOS+
Microglia/TNFa+
ACSA2+/iNOS+
ACSA2+/TNFa+
ACSA2+/IFNγ+
D
inflammatory microglia (% of singlets)
Control
RαMH
E
iNOS+ microglia (% ofsinglets)
Control
RαMH
F
EAE Control
EAE + RαMH
inflammatory microglia
Cluster-01 … Cluster-47
G
microglia (% of live cells)
CIA
CIA + PLX
CIA + RαMH
CIA + PLX + RαMH
****
H
CD11b+ Microglia
85.6%
CD11b
CD45

Supplemental Figure 6. (A) EAE clinical score of mice ± RαMH from day 12-14 post immunization. **(B)** Surface staining of cell populations in the CNS. **(C)** Intracellular staining of cell populations in the CNS. **(D)** Inflammatory microglia. **(E)** iNOS+ microglia. **(F)** Donut representation of the different cell clusters in the CNS after RαMH treatment. **(G)** Microglia from spinal cords 3 days after last PLX treatment. **(H)** CD11b+ MACS efficency of microglia analyzed by FACS. Data are expressed as the mean ± sd. Statistical difference was determined by One-way-ANOVA (A-C) and unpaired t test (D,E). *$p < 0.05$; **$p < 0.01$; ***$p < 0.001$. dpi = days post immunization, MACS = magnetic-activated cell sorting.

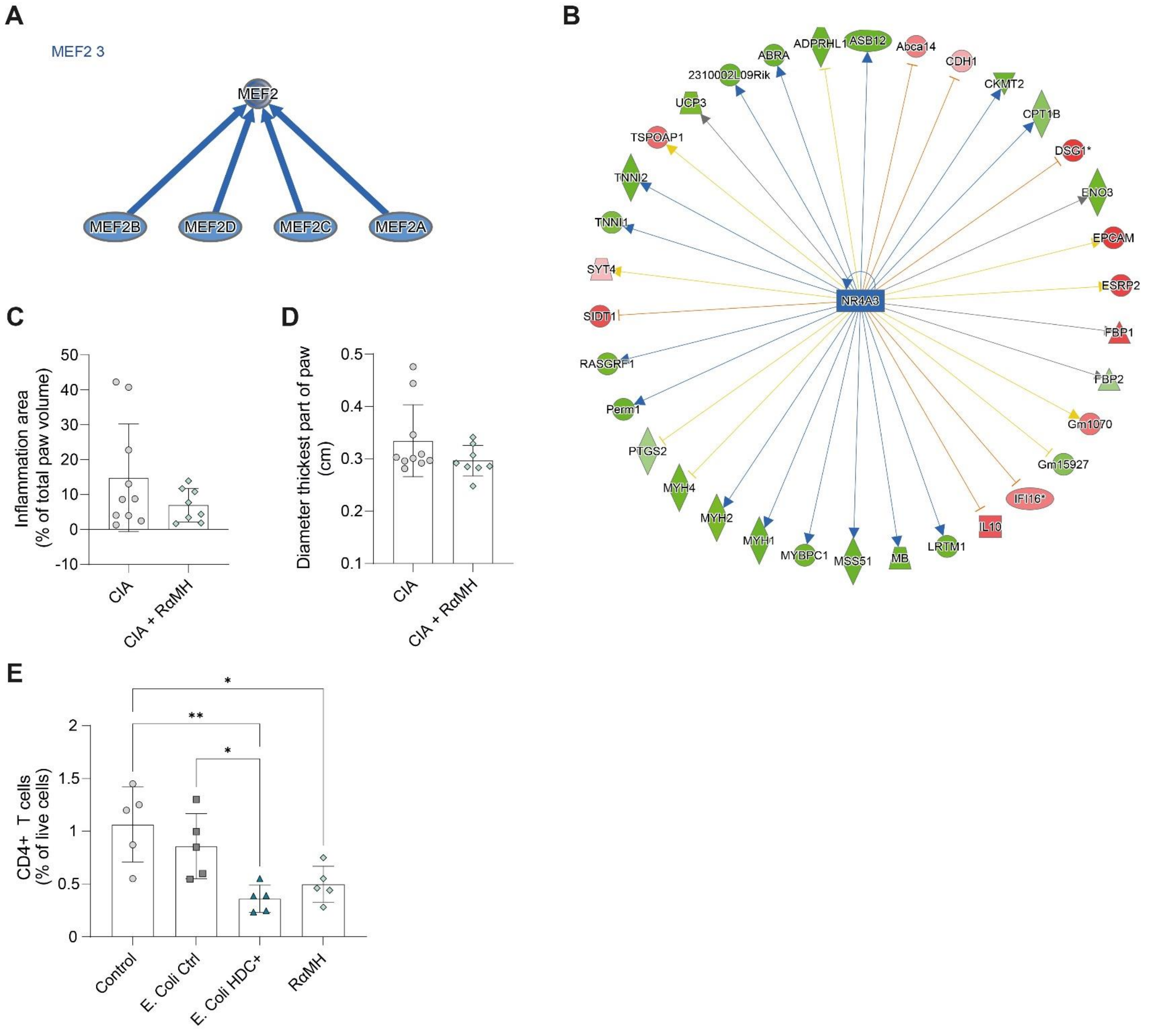

Supplemental Figure 7. (A) Causal Network of NR3A3 Neurovascular Coupling Pathway of RNAseq data from CD11b- cells from nervus plantaris (N.p.). **(B)** Causal network of MEF2 Neurovascular Coupling Pathway RNAseq data from CD11b- cells from nervus plantaris (N.p.). **(C)** Edema area calculated from MRI T2 STIR of hind paws (% of total paw volume) of CIA mice ± RαMH. **(D)** Diameter of the thickest part of the paws (cm) calculated from MRI of CIA mice ± RαMH. **(E)** FACS analysis of synovial CD4 T cells of CIA mice 8 days after last transfer of PBS (Control), E. Coli, HDC+ E. Coli or RαMH. Data are expressed as the mean ± sd. Statistical difference was determined by Student's t-test (C,D) and One-way ANOVA (E) . *p < 0.05; **p < 0.01;

Supplemental Methods:

Tissue isolation

Spleen/mLN/pLN

Tissue was isolated, minced and mashed through a pre-wetted 70 µm cell strainer into 5 ml PBS. The cell suspension was centrifuged at 1500 rounds per minute (rpm) for 5 minutes (min), the supernatant was discarded and the pellet was resuspended in 3 ml RBC lysis buffer and incubated for 5 min. The RBC lysis was stopped by adding 10 ml FACS buffer, the samples were centrifuged at 1500 rpm for 5 min and the supernatant was discarded. The pellet was resuspended in 5 ml FACS buffer and 100 µl of the suspension was transferred into a 96 well U-bottom plate for counting. According to the count each sample was adjusted to a concentration of 10^7 cells/ml in FACS buffer. For the cytokine-staining isolated cells were restimulated. Therefore, 10^6 cells were seeded into a V-bottom 96 well plate in 1X ReMed and incubated for 4 h at 37°C. All samples were washed with PBS, followed by viability staining for 30 min at 4°C in the dark with the Aqua NIR Fixable Viability Dye. After washing with FACS buffer the cells were incubated with a mix of extracellular binding antibodies (diluted in FACS buffer) and incubated for 30 min in the dark at RT. For intracellular staining, cells were washed with FACS buffer and fixed by using the FoxP3 Fixation and Permeabilization Kit. After fixation the samples were washed two times with permeabilization buffer and then incubated with 100 µl of the intracellular antibodies, diluted in permeabilization buffer, for at least 1 h in the dark. The samples were washed with permeabilization buffer and then reconstituted in FACS buffer until sample acquisition. Samples were acquired with a Beckman Coulter Cytoflex S or Cytek Northern Lights.

Spinal Cord and plantaris nerve

To isolate the cells from the PNS and CNS, mice were euthanized and perfused with cold 1× PBS. The nerves were isolated and collected on ice in 500µl digestion medium consisting of 0.15% Trypsin (Sigma-Aldrich, #T1426) diluted in medium and 0.3% (w/v) Collagenase II (Sigma-Aldrich, #11088858001). The tissue samples were mechanically diced in pieces using small microdissecting scissors and then shaken at 850 rpm for 10 minutes at 37 °C to facilitate enzymatic digestion. After trituration with a 1ml pipette tip, the samples were digested another 10 minutes on an orbital shaker at 850 rpm at 37 °C. The cell suspensions were then triturated with a 200µl pipette tip. Warm complete medium consisting of DMEM + GlutaMAX (Thermo Fisher Scientific Scientific, #61965026) supplemented with 10% FBS (Thermo Fisher

Scientific Scientific, #10438026) and 1% penicillin/streptomycin (Thermo Fisher Scientific Scientific, #10500064) was added to stop digestion. The homogenized tissue was then mechanically dissociated using a 5-ml serological pipette and triturated through a 100-µm cell strainer (Fisher Scientific, #22363548) into a fresh 15-ml conical tube. Following this, the samples were centrifuged at 400g for 7 minutes at 4 °C. After discarding the supernatant, the remaining mononuclear cell pellet was resuspended in 200 µl of 1x PBS and prepared for further applications. CD11b+ microglia were isolated from the cell suspension using the CD11b Microbead Isolation kit (Miltenyi, #130-049-601) according to the manufacturer's instructions.

Flow Cytometry

mLN, pLN, Spleen, Paws

Organs were harvested, and single-cell suspensions were prepared. For flow cytometric analyses, cells were stained with antibodies. Data was acquired on a Cytek Aurora Northern Lights (Cytek). For dimensionality reduction, samples were down-sampled using the DownSample plugin (version 3.3.1). Clusters were first calculated with phenograph (version 2.4). T-SNE reduction was done with Flowjo (version 10.8.1). Then, clustering was done with FlowSOM (version 3.0.18) using the phenograph cluster number as input.

Spinal Cord

For the characterization of CNS cells, we used flow cytometric analyses. The LIVE/DEAD™ Fixable Aqua Dead Cell Stain Kit (Thermo Fisher Scientific, #L34957) was used to differentiate between dead and viable cells according to the manufacturer's instructions. The cells were washed with cold 1x PBS and then incubated with Fc block (Purified Rat Anti-Mouse CD16/CD32; BD; #553141) in PBS for 10 minutes in the dark at room temperature. The cells were labelled with flow cytometry antibodies at 4°C in the dark for 30 minutes, diluted in FACS buffer (1x PBS, 2% FBS, 2 mM EDTA). After two washing steps in cold FACS buffer, the samples were resuspended in 1× PBS for acquisition. The following antibodies were used in the study: BV421-CD11b (Biolegend, #101235), BV480-CD11c (BD, #565627), BV570-Ly6C (Biolegend, #128029), BV605-CD68 (Biolegend, #137021), BV650-CD56 (BD, #748098), PE-eFlour610-CD140a (Thermo Fisher Scientific, #61140180), SuperBright780-MHCII (Thermo Fisher Scientific, #78532080), AF488-H3R (R&D, #FAB10200G), eFluor 450-CD3 (Thermo Fisher Scientific, #48003742), PE-Cy5-CD24 (Biolegend, #101811), PE-Cy7-CD31 (Thermo Fisher Scientific, #25031182), PerCP-eFlour710-CD86 (Thermo Fisher Scientific,

#46086280), PE-B220 (BD, #561878), PE-Cy5.5-CD45 (Thermo Fisher Scientific, #35045180), APC-Cy7-Ly6G (Biolegend, #127623), AF700-O4 (R&D, #FAB1326N), BUV737-CD154 (BD, #741735), AF660-CD19 (Thermo Fisher Scientific, #606019380), APC/Fire810-CD4 (Biolegend, #100479), APC-ACSA2 (Miltenyi Biotech; #130-117-386). For data analysis, the OMIQ platform was used. In more detail, cells were gated according to previously described methods 43,49. For dimensionality reduction, cells were downsampled to an appropriate number per group. Opt-SNE was performed (maximum 1000 iterations, perplexity 30, theta 0.5, verbosity 25) or UMAP (15 neighbors, minimum distance 0.4, 200 Epochs), followed by PhenoGraph clustering based on Euclidean distance. When comparing two groups, a two-class unpaired approach using SAM (Significance Analysis of Microarrays) was performed, with a maximum of 100 permutations and a False Discovery Rate (FDR) cutoff of 0.1.

µCT

µCT imaging was performed using the cone-beam Desktop Micro Computer Tomograph "µCT 40" by SCANCO Medical AG, Bruettisellen, Switzerland. The settings were optimized for calcified tissue visualization at 55 kVp with a current of 145 µA and 200 ms integration time for 500 projections/180°. For the segmentation of 3D-volumes, an isotropic voxel size of 8.4 µm and an evaluation script with adjusted grayscale thresholds of the operating system "Open VMS" by SCANCO Medical was used. Volume of interest tibia: The analysis of the bone structure was performed in the proximal metaphysis of the tibia, starting 0.43 mm from an anatomic landmark in the growth plate and extending 1.720 mm (200 tomograms) distally.

Histology

Tibial bones were fixed in 4% formalin for 24 h and decalcified in EDTA (Sigma-Aldrich). Serial paraffin sections (2 µm) were stained for tartrate resistant acid phosphatase (TRAP) using a Leukocyte Acid Phosphatase Kit (Sigma) according to the manufacturer's instructions. Osteoclast numbers were quantified using a microscope (Carl Zeiss) equipped with a digital camera and an image analysis system for performing histomorphometry (Osteomeasure; OsteoMetrics). Histological assessment was performed in a blinded manner by an independent investigator that was not involved in experimental treatment of the mice. Quantitative analysis of synovial inflammation, cartilage damage and bone erosion in the tarsal area was assessed with a microscope (Carl Zeiss) and the Osteomeasure software (OsteoMetrics, Decatur, GA), as described previously (1). In more detail, the area of synovial inflammation, was manually drawn in the tarsal

joints, including the calcaneocuboid, cuneonavicular, intercuneiform to tarsometatarsal joints (mean from at least two serial sections per animal). Corresponding quantitative data are given in mm^2.

SCFA measurement

Four to five replicates of frozen cecal samples (100 mg) or 50 µl of serum were weighed into a 2 ml polypropylene tube. The tubes were kept in a cool rack throughout the extraction. 33% HCl (50 µl for cecal contents or 5 µl for serum) was added and samples were vortexed for 1 min. One milliliter of diethyl ether was added, vortexed for 1 min, and centrifuged for 3 min at 4 °C. The organic phase was transferred into a 2 ml gas chromatography (GC) vial. For the calibration curve, 100 µl of SCFA calibration standards (Sigma) were dissolved in water to concentrations of 0, 0.5, 1, 5, and 10 mM and then subjected to the same extraction procedure as the samples. For GC mass spectrometric (GCMS) analysis, 1 µl of the sample (4–5 replicates) was injected with a split ratio of 20:1 on a Famewax, 30 m × 0.25 mm iD, 0.25 µm df capillary column (Restek, Bad Homburg). The GC-MS system consisted of GCMS QP2010Plus gas chromatograph/mass spectrometer coupled with an AOC20S autosampler and an AOC20i auto injector (Shimadzu, Kyoto, Japan). Injection temperature was 240 °C with the interface set at 230 °C and the ion source at 200 °C. Helium was used as carrier gas with constant flow rate of 1 ml/min. The column temperature program started with 40 °C and was ramped to 150 °C at a rate of 7 °C/min and then to 230 °C at a rate of 9 °C/min and finally held at 230 °C for 9 min. The total run time was 40 min. SCFA were identified based on the retention time of standard compounds and with the assistance of the NIST 08 mass spectral library. Full-scan mass spectra were recorded in the 25–150 m/z range (0.5 s/scan). Quantification was done by integration of the extracted ion chromatogram peaks for the following ion species: m/z 45 for acetate eluted at 7.8 min, m/z 74 for propionate eluted at 9.6 min, and m/z 60 for butyrate eluted at 11.5 min. GCMS solution software was used for data processing.

16s rRNA sequencing

For Figures 1 and 2 the following method was used: Genomic DNA was extracted from mouse feces using the ZymoBIOMICS DNA Miniprep kit, following the manufacturer's instructions. The 16S rRNA gene amplification of the V4 region (F515/R806) was performed according to a previously established protocol (2). Briefly, the DNA was normalized to 25 ng/µl and used for sequencing PCR, incorporating unique 12-base Golary barcodes via specific primers (obtained from Sigma). PCR was conducted in triplicates for each sample using Q5 polymerase (New England Biolabs),

with initial denaturation for 30 s at 98°C, followed by 25 cycles of 10 s at 98 °C, 20 s at 55 °C, and 20 s at 72 °C. After pooling and normalizing to 10 nM, PCR amplicons were sequenced on an Illumina MiSeq platform via 250 bp paired-end sequencing (PE250). The resulting raw reads were demultiplexed by idmp (https://github.com/yhwu/idemp) according to the specified barcodes. Libraries were processed including merging the paired-end reads, filtering low-quality sequences, dereplication to identify unique sequences, singleton removal, denoising, and chimera checking using the USEARCH pipeline version 11.0.667 [[PMID: 26139637]]. In brief, reads were merged using the fastq_mergepairs command (parameters: maxdiffs 30, pctid 70, minmergelen 200, maxmergelen 400), filtered for low quality with fastq_filter (maxee 1), and singletons were removed using the fastx_uniques command (minuniquesize 2). To predict biological sequences (ASVs,zOTUs) and filter chimeras, we used the unoise3 command (minsize 10, unoise_alpha 2) , following the amplicon quantification with the usearch_global command (strand plus, id 0.97, maxaccepts 10, top_hit_only, maxrejects 250). Taxonomic assignment was performed by Constax (classifiers: rdp, sintax, blast) utilizing the GreenGenes2 database [PMIDs: 33961008, 37500913], summarizing it into a biom-file for visualization in phyloseq (PMID: 23630581) and subsequent analysis.

For Supplementary Figures 1 and 2 the following method was used: Genomic DNA was extracted from mouse feces using the QIAamp PowerFecal Pro DNA Kit, following the manufacturer’s instructions. The 16S rRNA sequencing was kindly done by the working group of Dr. Stefan Wirtz, Universitätsklinikum Erlangen. 10 ng of stool genomic DNA was used in polymerase-chain reaction amplification of genomic 16S ribosomal RNA V4 regions using the prokaryotic primer pair (515F forward primer: 5'-GTGYCAGCMGCCGCGGTAA−3'; 806R reverse primer: 5'-GGACTACNVGGGTWTCTAAT−3') containing barcodes on the forward primer 515F (https://earthmicrobiome.org/protocols-and-standards/16s/). The NEBNext Q5 Hot Start Hifi PCR Master Mix (New England Biolabs, Frankfurt am Main, Germany) was used in a reaction employing 25 PCR cycles. The resulting PCR products were purified with AMPure XP Beads (Beckmann Coulter GmbH, Krefeld, Germany), pooled in equimolar ratios and analysed by 2 × 151 paired-end sequencing on an Illumina MiSeq device (Illumina Inc., San Diego, USA). Raw fastq files were then imported and analysed in QIIME2 v2024.10 with DADA2 as the method for quality control, dereplication and amplicon sequence variant (ASV) table generation. The SILVA small subunit database release 138 was used at a 99% similarity cut-off for taxonomic classification. For further analysis, ASV and taxonomic tables were

imported into R (version 4.2) as a phyloseq object and the "vegan" (2.6.4) package was used for diversity analysis and ordination. Before calculation of Bray-curtis and Jaccard (dis)similarities counts were log transformed for variance stabilization. For calculation of alpha diversity indices, repeated rarefaction to the smallest library size was performed. Ggplot2 was used for generation of graphical illustrations.

***E.coli* PCR**

E.coli DNA was amplified using the Microbial DNA qPCR Assay for Escherichia/Shigella spp from Qiagen. The amplified DNA fragments were loaded onto a 3% agarose gel.

Untargeted metabolomics of intestinal supernatant, serum and spinal cord extracts

<u>Sample preparation</u>

In order to extract analytes, stool suspensions of feces (in H_2O) were centrifuged at 16000 rpm and 4°C for 5 min using an Eppendorf 5427R centrifuge (Eppendorf; Hamburg, Germany). 100 µL of supernatant were mixed with 400 µL of ice-cold methanol containing recovery standards for evaluation of the quality of cell harvest and correction for variations. These standards were tridecanoic acid (25 µg/mL), DL-2-fluorophenylglycine (1.25 µg/mL), [2H6]-cholesterol (25 µg/mL), and DL-4-chlorophenylalanine (0.02 µg/mL). This was done for both HILIC and RP samples (see below). Before preparation for LC-MS analysis each sample was centrifuged at 16000 rpm and 4°C for 5 min using an Eppendorf 5427R centrifuge (Eppendorf; Hamburg, Germany). From each supernatant 350 µL were pipetted into two separate LC vials. 230 µL of each unknown sample were pooled to receive pooled QC samples. Of this pool 350 µL supernatant were pipetted into LC vials for each QC sample.

All samples were dried under a gentle stream of nitrogen at 30°C. Following to this, samples were reconstituted in 200 µL of eluent for HILIC analysis or eluent for RP analysis, to resemble conditions at the beginning of each chromatographic run (see next section for details). The eluents contained internal standards for evaluation of instrumental performance for each sample.

<u>LC-Orbitrap-MS-Analysis</u>

LC-MS analysis was performed as previously described (3). In brief, high-resolution mass spectrometry was done on an Exactive Focus Hybrid Quadrupole-Orbitrap mass spectrometer coupled to a Dionex Ultimate 3000 chromatographic

system (both from Thermo Fisher Scientific, Dreieich, Germany). Chromatographic separation was performed via both HILIC and RP chromatography. For HILIC separation, an Acquity UPLC BEH Amide, 1.7 µm, 2.1 x 100 mm column was used, while for RP an Acquity UPLC BEH C18, 1.7 µm, 2.1 x 100 mm column was installed. Both columns were equipped with a 2.1 x 5 mm guard column, respectively (all columns from Waters, Eschborn, Germany). The applied mass spectrometric settings, LC gradients, run times, QC limits, and batch sequences were identical to those previously shown by Gessner et al (3).

Data analysis

Data was analyzed with Compound Discoverer 3 (Thermo Fisher Scientific, Dreieich, Germany). Features were grouped and identified with a mass tolerance of 5 ppm and a retention time tolerance of 0.2 min. Differential analysis was performed using the log-transformed values for the peak area of a feature. P-values of ≤ 0.05 were considered as statistically significant.

Metabolite set enrichment analysis

Metabolite set enrichment analysis was performed using the MetaboAnalyst webtool (4). Only significantly increased level 1 annotated metabolites were used for this analysis.

Measurement of serum cytokines and chemokines (Legendplex)

Serum cytokines were measured with the LEGENDplex™ Mouse Proinflammatory Chemokine Panel (#740007, Biolegend) and LEGENDplex™ MU Th Cytokine Panel (12-plex) (#741044, Biolegend) following the manufacturer's instructions.

Ex vivo gut organ culture and c-FOS staining

Fabrication of the gut organ culture device and gut organ culture experiments were performed as previously described (5, 6). Briefly, intact whole colons were dissected sterilely from 14d old C57BL/6 mouse littermates reared under SPF conditions. The solid lumen content was gently flushed, and the gut fragment was threaded and fixed over the luminal input and output ports of the gut organ culture device, using sterile surgical thread. The culture device was placed in a custom-made incubator that maintains a temperature of 37°C, and tissue was maintained half-soaked in a constant flow of sterile medium (Iscove's Modified Dulbecco's Medium (IMDM) along with 20% Knockout serum, 2% B-27, 1% N-2, 1% L-glutamine, 1% non-essential amino acids, and 1% HEPES) using a syringe pump. Gut cultures were infused

with Histamine H3RA agonist at 250nM into the gut lumen using a syringe pump (littermate tissues infused with sterile medium served as an internal control). After 1 hour, tissues were collected for whole mount staining. Tissues were fixed with cold (4°C) 4% PFA for 1h. Tissues were then permeabilized at room-temperature using 0.5% triton for 2h and then transferred into blocking solution (0.1% triton, 5% BSA, and 10% donkey serum in PBS). Tissues were incubated with primary antibodies against beta-III-tubulin (pan-neuronal marker, Abcam, ab41489, 1:100) and c-Fos (Cell Signaling, cst2250s; 1:150), overnight at 4°C. Tissues were then washed and incubated with secondary antibodies (Cy3 (rabbit) and Cy5 (chicken), Jackson, 1:100) either for 2h, and then washed and counterstained with DAPI (1:1000) for 15 min. Colons were imaged using confocal fluorescence microscope (Leica Stellaris) and cFos nuclear localization was calculated using ImageJ. Briefly, ImageJ macro calculated nuclear c-Fos mean fluorescence intensity (MFI) in beta-III-tubulin+DAPI+ regions. The MFI values in individual nuclei were normalized to the average MFI in the internal control tissues, and statistical analyses were performed using GraphPad Prism 9 software.

RNAseq of SC and N. plantaris

RNA from CD11b+ and CD11b+ tissue suspension of SC and N. plantaris was isolated using the QiaGEN RNeasy Micro Kit following manufacturer's instructions. BulkRNAseq was performed by the Core Unit Next Generation Sequencing of the University Clinic Erlangen.

IPA

RNAseq data were further analyzed for enriched pathways and upstream regulators with the use of QIAGEN IPA (QIAGEN Inc., https://digitalinsights.qiagen.com/IPA) (7).

RNAseq of ileum

RNA from Ileal tissue samples from CIA mice ± oral RαMH treatment was isolated using the QiaGen RNeasy Mini Kit following the manufacturer's instructions. Novogene Sequencing – Europe (UK, Cambridge Sequencing Center) performed the Illumina RNA sequencing (RNAseq). In brief, sequencing libraries were generated using NEBNext® Ultra TM RNA Library Prep Kit for Illumina® (NEB, USA) and sequenced on an Illumina platform. Raw data (raw reads) of FASTQ format were processed through fastp. Mapping of the processed data to the reference genome Mus Musculus (GRCm38/mm10) was performed using the Spliced Transcripts Alignment to a Reference (STAR) software (8). FeatureCounts was used for the quantification of the mapped reads (9). Raw mapped reads were processed in R (Lucent

Technologies) with DESeq2 (10), to determine differentially expressed genes and generate normalized read counts. Pathway enrichment analysis was performed using the free online platform DAVID (11).

GSEA

Gene set enrichment analysis of RNAseq data was performed using the ClusterProfiler Package (Version 4.10.0) in R (12).

WesternBlot

For SDS-Page and Western Blotting the NuPAGE system from ThermoFisher Scientific was used. Tissues were homogenized in RIPA buffer containing proteinase and phosphatase inhibitors. Protein extracts were separated on a NuPAGE™ 4-12% Bis-Tris Protein Gels, transferred on a PVDC membrane and stained with antibodies against p38 (Cell Signaling #9218S) and phosphorylated p38 (Cell Signaling, #9211S). An antibody against β-Actin was used as loading control.

MRI imaging

In vivo imaging by MRI of the hind paws was performed at days 28 (before oral RαMH treatment) and 31 after CIA immunization (after 3 days of RαMH treatment) with a preclinical 7T MRI (ClinScan 70/30, Bruker BioSpin, Ettlingen, Germany) using a volume resonator: RF RES 300 1H 075/040 QSN TR. The imaging protocol included T1-weighted spin echo sequences, a short tau inversion recovery (STIR) sequence and a Dynamic Contrast Enhanced (DCE) measurement using a fast low-angle shot (FLASH) sequence (see Supplemental Table 1). During DCE-MRI the mice received an intravenous bolus injection of a low molecular weight gadolinium chelate agent (0.15 mmol/kg Gadobutrol, Gadovist, Bayer Vital GmbH, Leverkusen, Germany) over a time period of 10 s via a tail vein catheter.

MRI analysis

The total volume each hind paw was segmented on T2 STIR images. The volume of edema (bound tissue water) was quantified by segmenting all voxels in the hind paws with signal intensity above 5500 a.u. on STIR using a dedicated threshold-based OsiriX (aycan OsiriX PRO v. 2.08.006, aycan Medical Systems, LLC) Plugin, the Chimaera software (Chimaera GmbH Erlangen, Germany). DCE data was analyzed by using Bruker ParaVision 360 V3.2 and Microsoft Excel (Microsoft Office Professional Plus 2019).

Primary mouse astrocyte and microglia cultures and stimulation experiments

Brains of mice aged P0–P3 were dissected into PBS on ice. Brains of 6-8 mice were pooled, centrifuged at 500 × g for 10 min at 4 °C and resuspended in 0.25% Trypsin-EDTA (Thermo Fisher Scientific Scientific, #25200-072) at 37 °C for 10 min. DNase I (Thermo Fisher Scientific Scientific, #90083) was added at 1 mg/ml to the solution, and the brains were digested for 10 more minutes at 37 °C. Trypsin was neutralized by adding DMEM + GlutaMAX (Thermo Fisher Scientific Scientific, #61965026) supplemented with 10% FBS (Thermo Fisher Scientific Scientific, #10438026) and 1% penicillin/streptomycin (Thermo Fisher Scientific Scientific, #10500064), and cells were passed through a 70-µm cell strainer. Cells were centrifuged at 500 × g for 10 min at 4 °C, resuspended in DMEM + GlutaMAX with 10% FBS 1% penicillin/streptomycin and cultured in T-75 flasks (Sarstedt, #83.3911.002), pre-coated with 2 µg/ml Poly-L Lysine (PLL, Provitro, #0413) at 37 °C in a humidified incubator with 5% CO2 for 5–7 days until confluency was reached. Mixed glial cells were shaken for 30 min at 180 rpm, the supernatant was collected and the medium was changed, and then cells were shaken for at least 2 h at 220 rpm and the supernatant was collected and the medium was changed again. CD11b+ microglia were isolated from the collected supernatant using the CD11b Microbead Isolation kit (Miltenyi, #130-049-601) according to the manufacturer's instruction. For stimulation experiments, astrocytes and microglia were detached using TrypLE (Thermo Fisher Scientific, #12604013) and seeded in PLL-coated 48-well plates (Sarstedt, #NC1787625) at a density of 150.000 cells per well.

Histamine ELISA of serum and stool

For Histamine measurement in stool samples from mice, MS, RA and PsA patients, stool extracts were isolated from stool samples using the Stool preparation system, filled with extraction buffer IDK® Amino Extract (#K7999, ImmunDiagnostik). Histamine was measured using the Histamine Stool ELISA Kit (#K8213, ImmunDiagnostik) using the manufacturer's instructions and measured using a Tecan Sunrise Plate Reader.

Supplemental Table 1: Imaging Protocols for the different MRI used sequences

	T1 RARE Pre KM	T1 RARE Pre KM	T2 TurboRARE STIR + FS	DCE FLASH	T1 RARE Post KM + FS	T1 RARE Post KM + FS
Orientation:	Coronal	Sagittal	Sagittal	Coronal	Coronal	Sagittal
Echo Time:	6.5 ms	6.5 ms	33 ms	1.5 ms	6.5 ms	6.5 ms
Repetition Time	750 ms	750 ms	5512.8 ms	12.5 ms	828 ms	869.4 ms
Averages:	3	3	3	1	2	3
Scan Time:	4 m 48 s	3 m 59 s	8 m 49 s	8 m 18 s	3 m 32 s	3 m 42 s
Rare Factor:	2	2	7	7	2	2
Excitation Angle:	90°	90°	90°	15°	90°	90°
Refoc. Angle:	180°	180°	180°	180°	180°	180°
Image Size:	256 x 256	256 x 212	320 x 229	171 x 175	256 x 256	256 x 256
Field of View:	30x30 mm	30x 24.8 mm	35 x 25 mm	34.2 x 35 mm	30 x 30 mm	30 x 24.8 mm
Resolution:	0.117 x 0.117 mm	0.117 x 0.117 mm	0.109 x 0.109 mm	0.2 x 0.2 mm	0.117 x 0.117 mm	0.117 x 0.097 mm
Slice Thickness:	0.7 mm	0.7 mm	0.7 mm	1 mm	0.7 mm	0.7 mm
Slices:	40	42	42	3	40	42
Evolution	-	-	-	275 cycles	-	-

References

1. Zwerina J et al. TNF-induced structural joint damage is mediated by IL-1. Proc Natl Acad Sci U S A. 2007;104(28):11742-7.
2. Caporaso JG et al. Global patterns of 16S rRNA diversity at a depth of millions of sequences per sample. Proc Natl Acad Sci U S A. 2011;108 Suppl 1(Suppl 1):4516-22.
3. Gessner A et al. A Metabolomic Analysis of Sensitivity and Specificity of 23 Previously Proposed Biomarkers for Renal Transporter-Mediated Drug-Drug Interactions. Clinical Pharmacology & Therapeutics. 2023;114(5):1058-72.
4. Pang Z et al. MetaboAnalyst 5.0: narrowing the gap between raw spectra and functional insights. Nucleic Acids Research. 2021;49(W1):W388-W96.
5. Yissachar N et al. An Intestinal Organ Culture System Uncovers a Role for the Nervous System in Microbe-Immune Crosstalk. Cell. 2017;168(6):1135-48 e12.
6. Bootz-Maoz H et al. Diet-induced modifications to human microbiome reshape colonic homeostasis in irritable bowel syndrome. Cell Reports. 2022;41(7):111657.
7. Krämer A et al. Causal analysis approaches in Ingenuity Pathway Analysis. Bioinformatics. 2013;30(4):523-30.
8. Dobin A et al. STAR: ultrafast universal RNA-seq aligner. (1367-4811 (Electronic)).

9. Liao Y et al. featureCounts: an efficient general purpose program for assigning sequence reads to genomic features. (1367-4811 (Electronic)).
10. Love MI et al. Moderated estimation of fold change and dispersion for RNA-seq data with DESeq2. Genome Biology. 2014;15(12):550.
11. Huang DW et al. Systematic and integrative analysis of large gene lists using DAVID bioinformatics resources. Nature Protocols. 2009;4(1):44-57.
12. Yu G et al. clusterProfiler: an R package for comparing biological themes among gene clusters. Omics: a journal of integrative biology. 2012;16(5):284-7.
